# Attending to Marginalization in The Chronic Pain Literature: A Scoping Review

**DOI:** 10.1080/24740527.2024.2335500

**Published:** 2024-03-28

**Authors:** Laura Connoy, Michelle Solomon, Riana Longo, Abhimanyu Sud, Joel Katz, Craig Dale, Meagan Stanley, Fiona Webster

**Affiliations:** a Arthur and Sonia Labatt Family School of Nursing, Faculty of Health Sciences, Western University, London, Ontario, Canada; b Department of Family and Community Medicine, Temerty Faculty of Medicine, University of Toronto, Toronto, Ontario, Canada; c Department of Psychology, Faculty of Health, York University, Toronto, Ontario, Canada; d Department of Anesthesiology and Pain Medicine, Faculty of Medicine, University of Toronto, Toronto, Ontario, Canada; e Lawrence Bloomberg Faculty of Nursing, University of Toronto, Toronto, Ontario, Canada; f Tory Trauma Program, Sunnybrook Health Sciences Centre, Toronto, Ontario, Canada; g Western Libraries, Western University, London, Ontario, Canada

**Keywords:** chronic pain, marginalization, EDI-D

## Abstract

**Background:**

There has been a recent and, for many within the chronic pain space, long-overdue increase in literature that focuses on equity, diversity, inclusion, and decolonization (EDI-D) to understand chronic pain among people who are historically and structurally marginalized.

**Aims:**

In light of this growing attention in chronic pain research, we undertook a scoping review of studies that focus on people living with chronic pain and marginalization to map how these studies were carried out, how marginalization was conceptualized and operationalized by researchers, and identify suggestions for moving forward with marginalization and EDI-D in mind to better support people living with chronic pain.

**Methods:**

We conducted this scoping review using critical analysis in a manner that aligns with dominant scoping review frameworks and recent developments made to scoping review methodology as well as reporting guidelines.

**Results:**

Drawing on 67 studies, we begin with a descriptive review of the literature followed by a critical review that aims to identify fissures within the field through the following themes: (1) varying considerations of sociopolitical and socioeconomic contexts, (2) conceptual conflations between sex and gender, and (3) differing approaches to how people living with chronic pain and marginalization are described.

**Conclusion:**

By identifying strengths and limitations in the research literature, we aim to highlight opportunities for researchers to contribute to a more comprehensive understanding of marginalization in chronic pain experiences.

## Résumé

**Contexte**: La littérature mettant l’accent sur l’équité, la diversité, l’inclusion et la décolonisation (EDI-D) pour mieux comprendre la douleur chronique chez les personnes historiquement et structurellement marginalisées a récemment connu une augmentation, attendue depuis longtemps par de nombreux spécialistes de la douleur chronique.

**Objectifs:** À la lumière de cette attention croissante dans la recherche sur la douleur chronique, nous avons entrepris un examen approfondi des études portant sur les personnes vivant avec une douleur chronique et la marginalisation, afin de déterminer comment ces études ont été menées, comment la marginalisation a été conceptualisée et opérationnalisée par les chercheurs, et de recenser des suggestions pour aller de l’avant en gardant à l’esprit la marginalisation et l’EDI-D afin de mieux soutenir les personnes vivant avec la douleur chronique.

**Méthodes:** Nous avons mené cet examen de la portée en utilisant l’analyse critique, conformément aux cadres dominants et aux développements récents de la méthodologie de l’examen de la portée, ainsi qu’aux lignes directrices relatives aux rapports.

**Résultats:** En nous appuyant sur 67 études, nous commençons par un examen descriptif de la littérature, suivi d’un examen critique visant à déterminer les lacunes dans le domaine, en fonction des thèmes suivants : (1) les diverses interprétations des contextes sociopolitiques et socioéconomiques, (2) les confusions conceptuelles entre sexe et genre, et (3) les approches différentes concernant la manière dont les personnes vivant avec la douleur chronique et la marginalisation sont décrites.

**Conclusions:** En cernant les points forts et les limites de la littérature de recherche, nous visons à mettre en évidence les possibilités pour les chercheurs de contribuer à une meilleure compréhension de la marginalisation dans les expériences de douleur chronique.

**Abréviations:** TUO : trouble lié à l’utilisation d’opioïdes; DSM-5 : Manuel diagnostique et statistique 5; BPI : Questionnaire concis sur la douleur; NIDA : National Institute on Drug Abuse; IASP : Association internationale pour l’étude de la douleur; MTUO : Médicaments pour le trouble lié à l’utilisation des opioïdes; EIQ : Écart interquartile.

## Introduction

Interest in the experiences and management of chronic pain among marginalized groups is growing. Indeed, recognized global pain actors are dedicated to improving understandings of the implications of marginalization on chronic pain, whether through special interest groups,^1^ interprofessional pain curricula,^2^ or reports.^3^ This arguably aligns with the growing focus on equity, diversity, inclusion, and decolonization (EDI-D) in research more broadly, which aims to foster collaboration and safety and address discrimination and exclusion.^4,5^ For example, editors from leading pain journals, like the *Canadian Journal of Pain*, have recently endorsed principles aimed at addressing issues of inclusivity in pain science, scholarship, and publishing.^6^ The call for inclusivity has been echoed by researchers in the field.^7^ The focus on EDI-D and marginalization is of significant relevance for the field of chronic pain, as people living with inequity and discrimination (including due to racialized status and income) are more likely to live with chronic pain.^8–12^ Taking marginalization into account within the chronic pain space reflects an attempt to not only address issues of basic human dignity but also ensure that chronic pain research reflects the actual needs of people who are marginalized.

**Figure 1.**
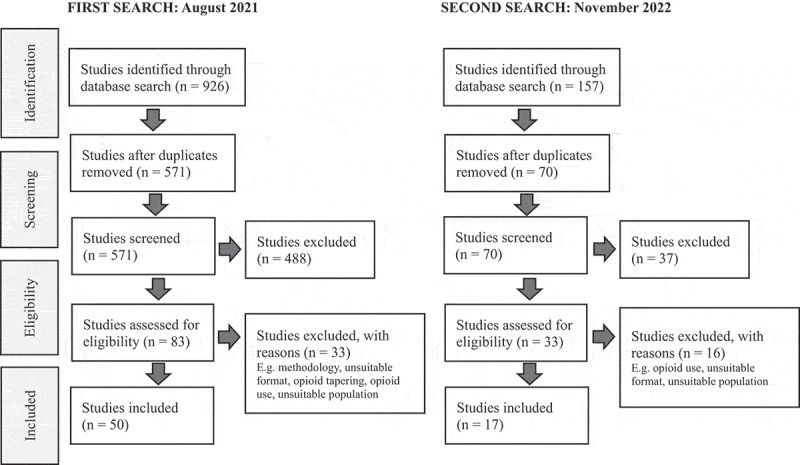
Preferred Reporting Items for Systematic Reviews and Meta-Analyses flow diagram of study selection process.

In light of these advancements, we undertook a scoping review that utilized a critical lens to inquire into studies that focus on people living with chronic pain and who are subjected to processes of marginalization. We did this to map how these studies were carried out, draw attention to how marginalization is conceptualized and operationalized within the literature, identify how people living within these contexts are discussed, and develop recommendations for this work going forward. Scoping reviews are useful for researching complex topics, such as chronic pain, and for mapping the literature for topics that have not yet been extensively reviewed,^13^ such as chronic pain within contexts of marginalization. Applying a critical lens to scoping reviews assists in mapping the literature by offering novel conceptual framings that may problematize assumptions and advance suggestions or solutions to chronic pain, “which is a crucial step toward imagining social change.”^14(p1468)^

Our guiding research question was, “What studies have been conducted that focus on people who live with chronic pain who are subjected to processes of marginalization?”^1^1Our original question was, “What studies have been conducted that engage with or focus on people who live with chronic pain and are subjected to processes of marginalization?” We found that this question caused some confusion among team members and so removed reference to “engage” for clarity. For transparency, we included “engage with” in the original research question to refer broadly to researchers who are engaging with the topic of marginalized groups. The broad scope of this term warranted slight revisions, which were accepted by the teaching and learning librarian (M.St.) who assisted with the search strategy and identification of databases. In answering our research question, we aimed to better understand how this specific subset of the chronic pain population has been discussed and how marginalization has been conceptualized within the chronic pain literature. By doing so, our goal was to identify gaps to assist the field in moving forward.

In this analysis, we extend the focus on marginalized groups to advance our understanding of marginalization as a *social process* that renders people/groups marginalized. Here, we are inspired by Young’s^15^ discussion of marginalization as stemming in and through unequal and oppressive power relationships that limit participation in social, political, cultural, and/or economic life. For example, within the realm of health care, marginalization can include sociopolitical rhetoric and health policies.^16^ The emphasis on marginalization as a social process maintains that people subjected to marginalization (often based on racialized status, sexual orientation, socioeconomic status, gender, ability, etc.) are not inherently deficient, nor victims (i.e., “marginal” or “marginalized”), but subjected to (and targeted by) social systems and structures that may be designed in ways that work against them. To engage with marginalization as a social process is to account for the oppression, deprivation, and structural injustice that are inflicted upon and impact peoples’ lives.^15^

## Methods

This scoping review is a COPE II Study project, which stands for “chronic pain ethnography.” Funded by the Canadian Institutes of Health Research, COPE II is a research program that focuses on chronic pain and marginalization by beginning in the standpoints of people struggling with marginalization.^17^ Specifically, COPE II uses the sociological approach of institutional ethnography^18^ to make visible the time and effort that go into living with chronic pain within broader contexts of struggle.^19^

We conducted the scoping review in a manner that aligns with the frameworks provided by Arksey and O’Malley^13^ and Levac et al.^20^ We also ensured that it reflected recent developments made to scoping review methodology,^21^ as well as those offered for reporting, such as PRISMA-ScR.^22^ We followed the five recommended steps in conducting a scoping review^20^: (1) identifying the research question, (2) identifying relevant studies, (3) study selection, (4) data charting, (5) summarizing and reporting results, and (6) consultation. We break these stages down below. The above guides do not include direction on how to conduct a scoping review through a critical lens. For guidance, we drew upon the work of others who have conducted a critical scoping review.^14^ We understand a critical approach as one that takes aim at the (re)discovery, explication, and improvement of social reality, including the forces that sustain inequity and injustice.^23^ By applying a critical lens to this scoping review, we set out to interrogate how marginalization has been discussed and conceptualized in the literature and offer recommendations for future research.

We partnered with a teaching and learning librarian (M.St.) in July 2021. We searched Medline, Embase, CINAHL, Sociological Abstracts, ERIC, and Scopus using the following terms related to chronic pain, marginalized groups, and research participation: “chronic pain,” “pain management,” “chronic pain management,” “chronic non-cancer pain,” “marginalized population,” “marginalized groups,” “vulnerable populations,” “marginalized persons,” “low income,” “drug users,” “racialized,” “ethnic groups,” “Indigenous Peoples,” “Indigenous,” “African Americans,” “homeless,” “homeless persons,” “addiction,” “substance-related disorders,” “mental health,” “mental illness,” “mental disorders,” “research subjects,” “patient participation,” “research participation,” “patient engagement,” “framework,” “lived experiences in research,” “shared decision making,” “patient engagement committee,” “community-based participatory research,” “health status disparities,” “participatory action research,” and “people with lived experiences.” These keywords were in alignment with our working concept of marginalization as noted above. By including the broad term “chronic pain” within the search strategy, we were prepared to receive results that spoke to varying diagnoses, mechanisms, and experiences. Incorporating this heterogeneity of chronic pain reflects our overall interest in the social aspects of chronic pain through the lens of marginalization. We could not assume a similar experience for those who, for example, have attributable causes for their chronic pain, as this would negate how the social (i.e., relations, organization, institutions, etc.) is foundational to experience.

The inclusion criteria were primary or secondary research approaches published in English that focused on chronic pain and marginalization. Primary and secondary sources were included because we were interested in the conceptualizations and operationalization of marginalization in the scientific literature in general. We excluded studies that focused on cancer pain and acute pain, as well as commentaries and abstracts. There were no date limits applied to the search. In total, 926 studies were imported into Covidence^24^ for screening, with 355 duplicates removed. This resulted in 571 studies for screening.

Titles and abstracts were independently and collaboratively screened in September 2021 by L.C., F.W., R.L., and M.S. We identified 83 studies that met the inclusion criteria, of which 3 were inaccessible. Between December 2021 and January 2022, the lead and senior authors (L.C. and F.W.) screened approximately 10% of the studies for relevance. The remaining studies were then divided among four authors (L.C., F.W., R.L., and M.S.) for full-text review, which was followed by data extraction. This screening of the 80 studies resulted in a total of 50 studies included in the scoping review. Reasons for exclusion at full-text review included methodological papers and attention to opioid tapering instead of chronic pain (Figure 1). From the 50 included studies, the same four authors then extracted key data elements, including participants, country, purpose, design, theory used, and findings. Each author carefully reviewed and took notes of the documents they were assigned. Then, during a virtual meeting, the authors discussed the data and identified themes. Extensive notes were taken of the meeting and the studies to create an audit trail. The team then organized the data into themes. The lead author (L.C.) then reread the entire data set to ensure accuracy.

We conducted an updated search in November 2022 to fill the gap from 2021 onwards. This resulted in 157 additional abstracts to screen in Covidence, with 87 duplicates removed. Upon review of the abstracts, 33 were included for full-text screening, and 17 of the full texts were included in the review (Figure 1). Members of the team (L.C., F.W., and M.S.) met virtually in May 2023 to discuss the new data and identify whether any new themes needed to be developed. Extensive notes were taken during the meeting and the studies to create an audit trail. The lead author then compared the themes from the second review with those of the first review. In total, 67 studies were included in this scoping review study.

The preliminary findings were presented to three researchers on the COPE II team (A.S., J.K., and C.D.), and we drew on their feedback in the consultation phase to enhance the relevance of our results and analysis. Although such forms of consultation are viewed as optional,^13,20^ this is an element of the scoping review that is useful and important^20,21,25,26^ given the multidisciplinary views they contributed from pain psychology, family medicine, and nursing. A final version of the manuscript was then reviewed by all authors. A review protocol was not published for this study.

## Findings

We have endeavored to present our findings (see Appendix A) in a way that speaks to the expectations of both traditional scoping reviews and scoping reviews that apply a critical lens. As a result, we divide our findings below into descriptive and critical syntheses. The categories that we developed for the descriptive review include populations, theory and frameworks, and primary purposes of the literature. Through critical analysis, we developed the following three interrelated themes: (1) varying considerations of sociopolitical and socioeconomic contexts, (2) conceptual conflations between sex and gender, and (3) differing approaches to how people living with chronic pain and marginalization are described. We use these themes to highlight what is missing or what requires ongoing work and to offer some suggestions for future research directions.^2^2These themes are not overly representative; not every reading can be included due to the large body of readings included in this scoping review.

### Descriptive Synthesis

All of the studies included in this scoping review (*n* = 67) were published between 1999 and 2022, with 46 published since 2018 (68.6%). Two-thirds of the studies were published in medical and clinical journals (*n* = 44, 65.6%), followed by a combined category of other journals (encompassing psychology, humanities, and inter-/multidisciplinary; *n* = 19, 28.3%) and four graduate dissertations (5.9%). The top representative journals were the *Journal of Pain* (*n* = 6, 8.9%) and *Pain Medicine* (*n* = 6, 8.9%), followed by the *Journal of General Internal Medicine* (*n* = 3, 4.4%), *PAIN* (*n* = 3, 4.4%), *Pain Management Nursing* (*n* = 2, 2.9%), *Global Qualitative Nursing Research* (*n* = 2, 2.9%), *Disability and Rehabilitation* (*n* = 2, 2.9%), and the *International Journal of Environmental Research and Public Health* (*n* = 2, 2.9%). The regional focus for the studies were the United States (*n* = 45, 67.1%), Canada (*n* = 7, 10.4%), Europe (Denmark, Spain, and Sweden, *n* = 5, 7.4%), Australia and/or New Zealand (*n* = 4, 5.9%), Singapore (*n* = 1, 1.4%), Vietnam (*n* = 1, 1.4%), and Canada and the United States (*n* = 1, 1.4%). Three studies were deemed not applicable in this regard. In terms of methods, 26 studies were qualitative (38.8%), 30 were quantitative (44.7%; which included randomized controlled/clinical trials), four were mixed-methods (5.9%), and seven were reviews (10.4%; including literature, narrative, meta-ethnography, and systematic).^16,27–32^ The most frequently cited method of data collection was interviews (*n* = 21, 31.3%), followed by questionnaires/surveys (*n* = 15, 22.3%). Most studies focused generally on chronic pain (*n* = 49, 73.1%), with the remaining focusing on specific chronic pain conditions, including migraine, fibromyalgia, sickle cell disease, diabetic neuropathy, HIV-related pain, irritable bowel syndrome, hip or knee osteoarthritis, and back, neck, or knee pain.

#### Populations

The populations included in the studies were sex workers, people who use drugs, people experiencing homelessness, people living with mental illnesses, veterans, migrants and refugees, elderly people, Indigenous Peoples, Black people, people of color (i.e., not Black and not Indigenous), women, people living with HIV, and people living with low income. Considering our focus on marginalization and EDI, we highlight those studies that exclusively focused on women, Indigenous Peoples, Black people, and people of colour, as these studies confront legacies of racism and sexism within chronic pain research. Within the studies included in this scoping review, 14 exclusively focused on women (20.8%),^32–45^ 12 exclusively focused on Black people (17.9%),^27,29,35,37,38,46–52^ three exclusively focused on Indigenous Peoples (4.4%),^53–55^ and six exclusively focused on people of color (8.9%).^42,45,56–59^

In the 12 studies that focused on chronic pain among Black people, some of the findings include the influence of environmental, socioeconomic, and racial influences on chronic pain^29^; lack of empathy and inadequate treatment within clinical spaces^47^; medical discrimination^37^; intergenerational empathy^50^; social and familial characterizations and expectations^38^; and pain coping.^51^ In the three studies that focused on chronic pain among Indigenous Peoples, emphasis was on the endorsement of traditional health practices within treatment programs^53^ and their placement within broader contexts of colonization,^54^ as well as the development of a culturally responsive online pain management program.^55^ Lastly, within the six studies that focused on chronic pain among people of color, some of the findings assessed the multiple dimensions of racialized discrimination^58^ and highlighted the importance of culturally relevant online health education interventions,^42^ culturally adapted physiotherapy,^57^ and holistic interventions for addressing pain in light of the role of adversity on pain experiences.^45^

#### Theories and Frameworks

A large portion of the studies did not specify a theory or guiding conceptual framework (*n* = 28, 41.7%). Of those studies that used a framework (*n* = 39, 58.2%), examples include Black feminist thought and critical arts-based inquiry,^35^ participatory action research,^55^ Gadamerian philosophical hermeneutics,^36^ two nested hierarchical models (Bronfenbrenner’s bioecological model and social ecological model),^16^ the National Institute on Aging Health Disparities Research Framework,^31^ Newman’s theory,^48^ the sociology of illness experience,^60^ Rhodes’ risk environment framework,^61^ Bronfenbrenner’s process–person–context–time model,^46^ Neuman’s systems model,^62^ the life course perspective,^45^ and the biopsychosocial model.^28,30,32,43,63,64^ We noted that studies that drew on theory offered more fulsome understandings of and approaches to marginalization, which sheds light on the structures underpinning aspects of chronic pain experiences among marginalized groups. For example, through the lens of Black feminist thought, Anthym^35(p18)^ powerfully illuminates “the interlocking oppressions of race, gender, and class experienced by Black women” while “center[ing] alternative sources of knowledge, as well as alternative methods of knowledge validation.” Nevertheless, only a minority of studies clearly offered a theoretical view of the processes of marginalization. Though some authors operationalized the terms “marginalization” or “marginalized” (*n* = 12, 17.9%), only two (*n* = 2, 2.9%) offered clear definitions.^16,35^

#### Primary Purposes of the Literature

We identified three primary purposes of the studies: 18 focused on the experiences of people living with chronic pain (26.8%; which can include, but are not limited to, discrimination and stigma); 17 focused on mental illness and/or substance use among those living with chronic pain (25.3%); and nine focused on intervention-focused research (13.4%). Combined, these three primary purposes represent 65.6% of the studies included in this scoping review (*n* = 44). We describe these below. Note that due to the large number of included studies, we are unable to provide a representative description. Instead, what is presented is a general overview of some of the studies with emphasis placed upon individual studies; review studies did not necessarily capture all of the studies included in this scoping review or analyze the data in a way that is similar to our approach.

##### Experiences of People Living with Chronic Pain

Focusing on the experience of living with chronic pain was the most common motive for the studies. Here, approaches to experience varied with regard to methodology and population. For example, Allen and colleagues^33^ conducted an exploratory qualitative analysis to understand the experiences of chronic pain among female survival sex workers in Vancouver’s downtown eastside. Through their work, they found that chronic pain may be understood as a symptom of the complexities of marginalization, which include trauma, mental illness, drug use, poverty, and stigma. Arman et al.^36^ aimed to understand women’s experiences of chronic pain through a Gadamerian philosophical hermeneutics approach and caring science and gender perspectives. Through their work, they highlighted the social and cultural impacts (including gender norms) on chronic pain. Booker and colleagues^49^ focused on experiences of osteoarthritis pain among aging African Americans through a qualitative descriptive design. They suggested the conceptual frame of “bearing the pain” to illuminate the process through which older African American adults live with pain, thereby offering insights into personal and cultural forms of self-management that have implications for care practices.

##### Mental Illness and/or Substance Use

The second most common research focus was mental illness and/or substance use among people living with chronic pain. For example, by focusing on experiences of chronic pain among those who use street opioids and/or cocaine, researchers^60^ were able to illustrate the “double stigmatization” faced by those living with chronic pain and labeled as “drug addicts.” This also shed important light on how “the chronic pain experience appeared to be a peripheral experience” in everyday life.^60(p4)^ Another study^65^ focused on hospitalized adults, which brought into focus trauma and homelessness and the barrier that pain presents to stopping drug use. Here, hospitalization and mortality were presented as motivational factors for change. In addition to the focus on drug/substance use, studies focused on mental illness (i.e., anxiety and/or depression) or a combination of mental illness and drug use. For example, Poleshuck et al.^44^ asserted through their quantitative work that comorbid pain and depressive symptoms are common among financially disadvantaged women. Naushad and colleagues^66^ aimed to understand the experiences and social consequences of living with depression and chronic pain, and their findings indicated higher levels of self-reported perceived stigma compared to those living with chronic pain or depression alone. Vogel and colleagues^67^ quantitatively examined the interdependence between chronic pain, substance use, and mental illness among individuals experiencing homelessness, suggesting an association between chronic pain and severity of substance use. Through these studies, we learn about the importance of accounting for other health factors, like drug/substance use and mental illness, in understanding and treating chronic pain.

##### Intervention-Focused Research/Studies

The final key focus of the included studies was intervention-focused, like health education programs, training programs, and pain management programs. For example, Bruns and colleagues^68^ examined the psychosocial experiences of low-income, ethnically diverse people living with pain before and after their participation in an integrative pain management program. Their findings indicated an increase in resilience and social connections through the uptake of new management strategies and perspectives, highlighting the importance of integrative medicine groups. Pagán-Ortiz and Cortés^42^ examined the acceptance of, and satisfaction with, an online health intervention for Spanish-speaking Latin women living with chronic pain. They paid specific attention to health literacy and empowerment, which highlights its feasibility and impact and the need for such interventions. Lastly, Perry et al.^55^ outlined their modified participatory action research framework in the development of a culturally relevant online pain management program with Māori in Aotearoa (New Zealand). Through this combined work, readers are made aware of how and in what ways communities can better understand and manage their chronic pain.

### Critical Synthesis

The above descriptive synthesis sheds light on important contributions made by researchers in the field by offering a “map” of the chronic pain terrain. In this section, our critical lens draws attention to risks and fissures within this literature.

#### Varying Considerations of Sociopolitical and Socioeconomic Contexts

To conduct research on marginalization necessitates acknowledgment of and engagement with the social (i.e., relations, organization, institutions, etc.). For example, one study presented different “systems” or models (such as sociopolitical models) to highlight the different factors that inform clinical conversations about chronic pain,^16^ and another employed a sociocultural level of analysis.^31^ Studies that accounted for social context were able to propose equity-oriented health care approaches,^10^ underline racialized discrimination^58^ and the role of neighborhood influences,^29^ highlight connections between pain and exile,^37^ and accentuate the misalignment between biomedical perspectives and the lived realities of women with chronic pain.^34^ Through these studies, which attempt to grapple with the sociopolitical and/or socioeconomic aspects of chronic pain, we learn of the complex historical and contemporary forms of inequity that are embedded within chronic pain experiences and treatments.

In the 67 studies included in this scoping review, 19 offered little to no engagement with the social aspects of chronic pain (28.3%); the lack of attention to, and engagement with, social aspects in the broader literature was noted in one study.^31^ For example, exploring the relationship between stressors (a term that focuses on the person) and the quality of life of people living with chronic pain can lead authors to emphasize individual solutions, such as offering time management strategies.^62^ Though perhaps a useful downstream tool at the individual level, time management on its own is insufficient to address the upstream forces that sustain processes of marginalization, which might be better addressed by calling for affordable and adequate social supports to assist in the management of pain.

The conceptualization of the sociopolitical and socioeconomic aspects of chronic pain was not clear or consistent within the collected literature. We noted references to marginalization/marginalized, disparity, underrepresented, undertreated, perceived injustice, and vulnerability, yet little to no definitions were provided, and many were often used interchangeably. Without conceptual clarity, researchers risk misinterpreting the realities of life in a social world and conflating conceptually distinct terminology. Furthermore, conceptual clarity and consistency ensure that researchers stay focused on the broader social issues that inform chronic pain experience and treatment.

There are risks, however, that even with sustained attention to groups who have been marginalized, researchers may (unwittingly) suggest individual solutions like acupuncture^69^ or develop interventions that target “exercise, psychological wellbeing, regaining function, emotional wellbeing, sleep hygiene, and stress management”^42(p12)^ that do not necessarily take social barriers into account.^49,55^ Though authors reported some modest positive effects of these interventions, it is questionable whether such individualized solutions are sufficient for addressing complex health and social issues that drive marginalization.

#### Conceptual Conflations between Sex and Gender

We noted how gender rather than sex was often not accounted for in most studies and that it was often not clearly defined. Indeed, only two definitions of gender were provided: gender is “a social structure, interwoven with reproductive processes, identity, and power that differs between cultural and historical contexts”^36(p773)^ and gender is a social construct, “refer[ring] to social and cultural expectations, beliefs, and norms” that are “contextualized and recurrently created though social interactions.”^64(p2)^ Without clear definitions of gender, researchers may miss how it is socially constructed, may employ gender descriptively, and/or may conflate it with sex.

As noted by some of the authors included in this scoping review, women face disparities within the field of chronic pain care,^32^ and these can stem from the social construct of gender.^34,36,37^ We anticipated a high percentage of studies that would take into account women’s gendered experiences of chronic pain, but there was limited exclusive focus on women within the studies included in this scoping review (*n* = 14, 20.8%). Only six of these 14 studies focusing on chronic pain in women referred to gender with a clear distinction from sex (42.8%),^32,35–38,40^ and three failed to incorporate it at all (21.4%).^39,44,45^ We found this to be a surprising finding. Many of the chronic pain conditions faced by women, like fibromyalgia, for example, are often not effectively managed or understood, which, according to one study, necessitates a “gender-sensitive perspective.”^34(p235)^ Attending to gender can allow researchers to better account for those socially constructed aspects of chronic pain that affect women, like (paid and unpaid) work and overperformance, biases, family life and obligations (i.e., caregiving), embodiment, and other struggles and life situations.^34,36^ Doing so may also assist in illuminating processes of marginalization as they pertain to women living with chronic pain. More research is needed that accounts for gender and women’s gendered experiences of chronic pain.

However, as argued within two studies included in this review,^37,38^ it is not enough to simply consider gender. Gender is but one social factor that shapes experiences of chronic pain; it intersects with and is intersected by various other social identities.^37^ For example, though Dugan et al.^40^ called attention to the limited engagement with gender in analyses of discrimination and pain, over half of their respondents were white and highly educated, which yields a highly specific and skewed understanding of the role of gender in the lives of a specific group of women. Of the 14 studies focusing exclusively on women,^33^ six took into account how gender intersects with racialized identity (42.8%),^35,37,38,42,45^ with three of these studies focusing on Black women^35,37,38^ and two focusing on Latinx women.^42,45^ Four studies either included or made reference to the need for a non-binary understanding of gender.^10,61,68,70^

#### How People Living with Chronic Pain and Marginalization Are Described

We took note of how people living with chronic pain and marginalization were described, including diversity in the use of person-first language. Person-first language (person precedes disability, such as “persons living with chronic pain”) was prominent in 37 studies (55.2%). Identity-first language (disability precedes person, such as “chronic pain patient”) was prominent in 13 studies (19.4%); both were prominent in 17 studies (25.3%). We also noted some contentious language use. Examples include “impoverished medical populations,”^71^ “impoverished minorities,”^29^ “indigent adults,”^48^ and “vulnerable patients,”^16,68,72^ which may situate blame with the person living with pain and perpetuate harmful stereotypes. Though the term “participant” was dominant, this sometimes occurred alongside the terms “subject” and “patient,” which can also individualize and medicalize and strip away the identities of people living with pain. There were also references to “drug abuse”^47,48,73,74^ and “substance abuse,”^28,51,63,74,75^ which are terms laden with negative connotations, stigma, and individual blame. Other references of concern include “resilience,” “capacity,” and “empowerment,” which are individualized notions that may obscure systemic and structural issues that also contribute to chronic pain.

We noted that in many studies researchers often employed broad, sweeping categories, such as “women,” “African Americans,” “Hispanics,” or “American Indian people.” Focusing on experiences of chronic pain within these populations is needed, yet such flattened categorizations may sustain a problematic assumption of a universal experience of living with chronic pain that is void of complex social contexts. As a result, researchers may be prevented from gaining adequate insights into the complexities defining chronic pain and marginalization and the diversity of experiences within groups.

## Discussion

This scoping review provides an account of the contributions made by researchers to understanding chronic pain within marginalized settings. It also calls attention to fissures. We draw on these findings to provide some direction on future research on chronic pain and marginalization, specifically, conceptual clarity, the social aspects of experience, and language use. Given the relative lack of shared conceptual understandings and definitions of marginalization as a social process, we argue that theoretical approaches grounded in the social sciences—especially with critical leanings—could offer new and innovative ways forward in the field. Indeed, editors from leading pain journals like the *Canadian Journal of Pain* have endorsed the use of “social frameworks for interpretations” in their proclamation to eliminate disparities and support inclusive environments in pain science, scholarship, and publishing.^6(p3)^

It has recently been asserted that we are witnessing an “emergent” generation of pain disparities research that is rooted in justice.^76^ To usher in this generation, one immediate action is to “re-shape our thinking” about chronic pain via new and/or different conceptualizations and re/definitions.^76(p6)^ Recent work undertaken by our team underscores the importance of introducing new conceptualizations, like the concept of chronic struggle, to provide nuance to understandings of largely biological notions of chronic pain when describing the experiences of people with chronic pain who face marginalization.^19^ To move toward new and/or different conceptualizations and re/definitions, we must be aware of the existing need for conceptual clarity and careful application, as made evident in this scoping review and by others.^77^ Simply adopting concepts, like marginalization, for example, without clear definition can perpetuate conceptual imprecision and/or inaccuracies in practical applications. This, in turn, can hinder innovative insights and interventions that draw attention to social and structural issues.

One of the key findings of this scoping review is the lack of clear definitions. However, even when definitions are provided, they may not necessarily call into question the embedded assumptions that underpin them. For example, though the terms “vulnerability”, “resilience”, and “empowerment” are prominent in chronic pain scholarship and evident within this scoping review, they may direct our focus to the individual, which steers attention away from critically analyzing the roles of social systems, structures, and relations in people’s lives. To be clear, when authors incorporate such terms, they are not defining them or intending to use them in ways that negatively characterize or impact people living with chronic pain. However, the individualized aspects of terms that focus on the biological or psychological may unintentionally facilitate adverse effects or outcomes by perpetuating stigma and/or assumptions of inherent weakness or deficiency.^78,79^ It is in this regard that the use of these terms requires care and caution, particularly when applied within contexts of marginalization. Based on our findings, the employment of “vulnerability” and other abovementioned concepts in chronic pain research set within contexts of marginalization may be less useful because they may risk obscuring systemic and structural barriers that inform chronic pain and sustaining individual blame. Bringing in a focus on the social aspects of chronic pain—for example, through the concept of marginalization—can be complementary and offer opportunities to further explore the mechanisms that lead to some groups facing greater barriers to care and worse outcomes.

In keeping with our focus on language, person-first language was prominent in over half of the studies included in this scoping review. This is indeed a contentious aspect of communication.^80^ Some argue that person-first language may sustain stigma, because it is typically only used with people living with “undesirable” conditions as compared to those without.^81^ In contrast, others assert a need for person-first language to assist with destigmatization.^82^ We support the use of person-first language within the chronic pain context, which is also reflected in leading pain journals.^6^ Person-first language supports the calls of people living with pain to be acknowledged as such—as people with specific and unique social experiences.^83^ With recent calls for EDI in pain science, which includes the use of “language that is inclusive and minimizes bias,”^6(p2)^ we urge researchers to be more attentive to how they refer to people living with chronic pain and marginalization.

Our focus on gender highlighted a paucity of studies that accurately engage with this concept, which has been noted by others as a key challenge in pain research.^84^ This is in light of calls for both gender-sensitive research^34^ to account for the influence of gender on women’s experiences of chronic pain^85–88^ and greater critical awareness of the “gender paradox of pain,” defined as the “persisting gender biases in health care and their seeming acceptance.”^32(p491)^ Calls to incorporate gender as an analytical lens are also supported by national funding bodies in the health sciences, including those in Canada and the United States of which 79% of the studies included in this scoping review are based.^89,90^ We support such calls, especially with regard to chronic pain in women. Women are more affected by chronic pain than men,^91,92^ yet many of the chronic pain conditions most experienced by women are poorly understood, stigmatized, underfunded, and underresearched.^93,94^ Accounts of illness are shaped by gender discourses, which can sustain skepticism, rejection, and/or disregard.^88,95^ A gendered approach to chronic pain can shed light on the sociopolitical aspects of this disease, allowing for better understandings and treatments.

However, it is important to approach gender with care. There are risks in preserving a binary gender division,^10^ which can sustain pain disparities among gender-diverse people who are at greater risk of discrimination or denial of care within health care settings.^96^ It is for this reason that health professions’ education must attend to the needs and concerns of the 2SLBGTQIA+ community.^97,98^ A related risk is the denial of heterogeneity, intersecting identities, and oppressions.^38,99,100^ Accounting for the ways in which racialized identity, socioeconomic status, geographic location, ability, sexual identity, and other categories intersect with gender would provide nuanced insights into experiences of chronic pain.^101^ Yet, in this scoping review, we noted how intersections of gender and racialized status have yet to be sufficiently addressed in chronic pain scholarship. Racialized populations face inequitable barriers when it comes to pain treatment and management,^102–105^ and illuminating how racialized status intersects with gender is necessary to further advance our understandings of these inequities. Researchers caution against relying on biological restrictions of race that can sustain a separation from the social—namely, racism.^106^ As argued by Hood et al.^107(p922)^ “When we do not examine racism and its effects, troubling assertions of unmeasured biological or genetic reasons for racialized differences in pain outcomes can occur.” This scoping review points to a need for socially grounded scholarship that can account for intersecting identities and multiple and intersecting sources of oppression and inequity. This review also suggests a need for antiracism research practices in chronic pain scholarship^108^ and other similar research practice recommendations, such as the use of EDI-D frameworks.

## Recommendations for Future Studies on Chronic Pain and Marginalization

The aim of this scoping review was to map out how studies of chronic pain and marginalization were carried out, how people living with chronic pain who are subjected to marginalization are imagined and discussed, and how marginalization is conceived within the literature. In doing so, we hoped to identify valuable contributions and lingering gaps to assist the field in moving forward. Below, we detail some potential strategies for future research on chronic pain and marginalization. These are intended to complement those strategies already offered by others in the field.^76^ Based on this scoping review, we offer the following interrelated suggestions:

1. Offering clear accounts of sociopolitical and socioeconomic contexts of chronic pain may help to extend understandings beyond individual-level clinical explanations, which is pertinent within contexts of marginalization. This approach is often found within social science literatures and critical forms of scholarship that account for the broader social influences that inform people’s lives, like social norms, language use, and sociopolitical conditions. Including and working with social scientists and critical scholars when conducting chronic pain research could also help to achieve this.^3^3For example, our COPE II team has been recognized by the Canadian Institutes of Health Science (CIHR) for their ability to address research challenges, like the social aspects of chronic pain, through an interdisciplinary team makeup that blends psychology, medicine, nursing, anthropology, and sociology.^109^ Without an account of the social aspects that inform the chronic pain experience, researchers risk rendering people responsible for their experiences by disregarding pre-existing and often long-standing barriers and inequities.
2. Future research could provide clear definitions and explanations of terminology and guiding theoretical or conceptual frameworks. Others have similarly discussed issues regarding conceptualizations in the chronic pain space.^77,110^ Additionally, we note the importance of describing people/participants not in broad, homogeneous categories but rather in ways that account for intersecting identities, which has also been noted by others.^111^ Addressing these issues offers transparency, reduces conceptual conflation, and avoids unintentionally harmful concepts or biased language. The safety and dignity of people living with chronic pain and marginalization are among the cornerstones of EDI-D, in which language plays a key role.
3. Accounting for the role of gender in research may better prepare researchers to explicate the nuances of chronic pain and marginalization. By “gender,” we are referring to the social construct that includes socially and culturally ascribed norms, roles, expectations, identity, and expression that—for the purposes of this study—shape experiences of pain (such as how it is recognized and treated).^85,112,113^ It is different from the biologically and physiologically rooted concept of “sex,” yet it often remains conflated in the literature. Utilizing a gender lens assists in highlighting the inequity, discrimination, and dismissal that, for example, women with chronic pain may face in their daily social and clinical encounters.^86–88^ It can also shed important and much-needed light on the unique experiences of chronic pain among gender-diverse people. Though gender is a defining element of health, it is one of many social aspects defining peoples’ lives. Incorporating an intersectional theoretical lens may be one means to avoid homogeneous discussions of populations, which can flatten understandings of chronic pain, by illuminating how, for example, gender, racialized status, and socioeconomic status influence chronic pain experiences. Other recommendations for future research based on our findings, and those of others,^84^ include the incorporation of clear and accurate definitions of gender and interrogating potentially harmful and exclusionary assumptions of gender.

## Limitations

We are aware of the limitations of language and its changing nature. We may have included terms that may not be used by all researchers or groups, or we may have missed terms that are only newly emerging. We also note that by using the guiding concept of marginalization, we may have missed research that does not employ this concept or other synonymous concepts. We acknowledge the risk of applying a term that might not be accepted by all groups or individuals. Lastly, our inclusion of chronic pain as a search strategy yielded varying diagnoses, mechanisms, and experiences. The incorporation of this term was intended to capture all aspects of chronic pain through the lens of marginalization. However, this may have also influenced our findings, as certain chronic pain conditions (i.e., arthritis) have greater support within medical frameworks, leading to a significantly different experience than those living with conditions that have less support (i.e., fibromyalgia).^114^ We do not want to assume that social aspects do not play a role within this chronic pain hierarchy, and it is for this reason that the broad use of the term “chronic pain” was applied within the search.

## Conclusion

Increasing attention is being paid to the role of EDI-D frameworks as they pertain to people who are historically and structurally marginalized and living with chronic pain. Doing so renders visible the diverse social aspects that come to define and shape experiences of chronic pain. However, understandings of what constitutes marginalization and how it is approached within the chronic pain literature vary. This review identified that (1) greater conceptual clarity and application of marginalization within chronic pain research is required, (2) the inclusion of social aspects is necessary to make visible processes of marginalization and thier effects on chronic pain, and (3) there is a need to pay attention to how biases may unintentionally enter research studies through language and generalizing references to heterogeneous populations. We hope our findings and suggestions will be of assistance to researchers who study marginalization and chronic pain, especially as it pertains to the complexity of experience (beyond the individual) as it is located within systems and structures.

## Supplementary Material

Supplemental Material

Supplemental Material
