# Supplementary material for: Attending to Marginalization in The Chronic Pain Literature: A Scoping Review
Source: Can J Pain. 2024 Mar 28;8(2):2335500. doi: 10.1080/24740527.2024.2335500 (PMC11146439; doi:10.1080/24740527.2024.2335500)
Supplement: Supplemental Material [file UCJP_A_2335500_SM2790.docx]

**Attending to marginalization in the chronic pain literature:
A scoping review**

Laura Connoy, Michelle Solomon, Riana Longo, Abhimanyu Sud, Joel Katz, Craig Dale, Meagan Stanley, and Fiona Webster

Laura Connoy (corresponding author)

Arthur and Sonia Labatt Family School of Nursing, Faculty of Health Sciences, Western University, London, Ontario, Canada

Twitter: @LauraConnoy

lconnoy@uwo.ca

Michelle Solomon

Arthur and Sonia Labatt Family School of Nursing, Faculty of Health Sciences, Western University, London, Ontario, Canada
Twitter: @MichelleSSolo

msolomo7@uwo.ca

Riana Longo

Arthur and Sonia Labatt Family School of Nursing, Faculty of Health Sciences, Western University, London, Ontario, Canada

Twitter: @rianalongo

rlongo5@uwo.ca

Abhimanyu Sud

Department of Family and Community Medicine, Temerty Faculty of Medicine, University of Toronto, Toronto, Ontario, Canada

Twitter: @doc_sud

abhimanyu.sud@utoronto.ca

Joel Katz

Department of Psychology, Faculty of Health, York University, Toronto, Ontario, Canada

Department of Anesthesiology and Pain Medicine, Faculty of Medicine, University of Toronto,

Toronto, Ontario, Canada

Twitter: @joeldkatz

jkatz@yorku.ca

Craig Dale

Lawrence Bloomberg Faculty of Nursing, University of Toronto, Toronto, Ontario, Canada

Tory Trauma Program, Sunnybrook Health Sciences Centre, Toronto, Ontario, Canada

Twitter: @craig_dale1

craig.dale@utoronto.ca

Meagan Stanley

Teaching and Learning Librarian, Western University, London, Ontario, Canada

mstanle6@uwo.ca

Fiona Webster

Arthur and Sonia Labatt Family School of Nursing, Faculty of Health Sciences, Western University, London, Ontario, Canada

Twitter: @FionaWebster1

fiona.webster@uwo.ca

**Abstract**

Background: There has been a recent and, for many within the chronic pain space, long overdue increase in literature that focuses on the role of equity, diversity, inclusion, and decolonization (EDI-D) in understanding chronic pain among people who are historically and structurally marginalized. Aims: In light of this growing attention in chronic pain research, we undertook a scoping review of studies that focus on people living with chronic pain and marginalization in order to map how these studies were carried out, how marginalization was conceptualized and operationalized by researchers, and to identify suggestions for moving forward with marginalization and EDI-D in mind to better support people living with chronic pain. Methods: We conducted the scoping review using critical analysis in a manner that aligns with dominant scoping review frameworks and reflects recent developments made to scoping review methodology, as well as those offered for reporting. Results: Drawing on 67 studies, we begin with a descriptive review of the literature followed by a critical review that aims to identify fissures within the field via the following themes: 1) varying considerations of socio-political and socio-economic contexts; 2) conceptual conflations between sex and gender; and 3) differing approaches to how people living with chronic pain and marginalization were described. Conclusion: By identifying strengths and limitations in the research literature we aim to highlight opportunities for researchers to contribute to a more comprehensive understanding of marginalization and EDI-D in chronic pain experiences.

Key words: chronic pain, marginalization, EDI-D

**Introduction**

Interest in the experiences and management of chronic pain among marginalized groups is growing. Indeed, recognized global pain actors are dedicated to improving understandings of the implications of marginalization on chronic pain, be it through special interest groups,^1^ interprofessional pain curricula,^2^ or reports.^3^ This arguably aligns with the growing focus on equity, diversity, inclusion, and decolonization (EDI-D) in research more broadly, which aims to foster collaboration and safety, and address discrimination and exclusion.^4,5^ For example, editors from leading pain journals, like *Canadian Journal of Pain*, have recently endorsed principles aimed to address issues of inclusivity in pain science, scholarship, and publishing.^6^ The call for inclusivity has been echoed by researchers in the field.^7^ To be sure, the focus on EDI-D and marginalization is of significant relevance for the field of chronic pain where people living with inequity and discrimination (including due to racialized status and income) are more likely to live with chronic pain.^8-12^ Taking marginalization into account within the chronic pain space reflects not only an attempt to address issues of basic human dignity, but also ensure that chronic pain research reflects the actual needs of people who are marginalized.

In light of these advancements, we undertook a scoping review that utilized a critical lens to inquire into studies that focus on people living with chronic pain who are subjected to processes of marginalization in order to: map how these studies were carried out, draw attention to how marginalization is conceptualized and operationalized within the literature, identify how people living within these contexts are discussed, and develop recommendations for this work going forward. Scoping reviews are useful for researching complex topics, such as chronic pain, and for mapping the literature for topics that have not yet been extensively reviewed,^13^ such as chronic pain within contexts of marginalization. Applying a critical lens to scoping reviews assists in building on such mappings of the literature by offering novel insights through conceptual framings, problematizing assumptions, and advancing suggestions or solutions to chronic pain, “which is a crucial step toward imagining social change.”^14(p.1468)^

Our guiding research question was: What studies have been conducted that focus on people who live with chronic pain who are subjected to processes of marginalization?^[[1]](#footnote-2)^ In answering our research question, we aimed to better understand how this specific subset of the chronic pain population has been discussed within chronic pain research, and how marginalization has been conceptualized within the chronic pain literature. To further the contributions offered by researchers studying chronic pain within contexts of marginalization, our goal was to identify gaps in order to assist the field in moving forward.

In this analysis, we extend the focus on marginalized groups to advance our understanding of marginalization as a *social process* responsible for rendering people/groups as marginalized. Here, we are inspired by Young’s^15^ discussion of marginalization as stemming in and through unequal and oppressive power relationships that limits participation in social, political, cultural and/or economic life. For example, within the realm of healthcare, marginalization can include sociopolitical rhetoric and health policies.^16^ The emphasis on marginalization as social process maintains that people subjected to marginalization (often based on racialized status, sexual orientation, socioeconomic status, gender, ability, etc.) are not inherently deficient, nor victims (i.e., ‘marginal’ or ‘marginalized’), but subjected to (and targeted by) social systems and structures that may be designed in ways that work against them. To engage with marginalization as a social process is to account for the oppression, deprivation, and structural injustice that is inflicted upon and impacts upon peoples’ lives.^15^

**Methods**

The scoping review is a COPE II Study project, which stands for chronic pain ethnography. Funded by the Canadian Institutes of Health Research, COPE II is a research program that focuses on chronic pain and marginalization by beginning in the standpoints of people struggling with marginalization.^17^ Specifically, COPE II uses the sociological approach of institutional ethnography (IE)^18^ to make visible the time and effort that goes into living with chronic pain within broader contexts of struggle.^19^

We conducted the scoping review in a manner that aligns with the frameworks provided by Arksey and O’Malley^13^ and Levac et al.^20^ We also ensured that it reflected recent developments made to scoping review methodology,^21^ as well as those offered for reporting, such as PRISMA-ScR.^22^ We followed the five recommended steps in conducting a scoping review:^20^ (1) identify the research question, (2) identify relevant studies, (3) study selection, (4) data charting, (5) summarizing and reporting results; and, (6) consultation. We break these stages down below. The above guides do not include direction on how to conduct a scoping review through a critical lens. For guidance, we drew upon the work of others who have conducted a critical scoping review.^14^ We understand a critical approach as one that takes aim at the re/discovery, explication, and improvement of social reality, including the forces that sustain inequity and injustice.^23^ By applying a critical lens to the scoping review, we set out to interrogate how marginalization was discussed and conceptualized in the literature, and offer recommendations for future research.

We partnered with a Teaching and Learning Librarian (MSt) in July 2021. We searched Medline, Embase, CINAHL, Sociological Abstracts, ERIC, and Scopus using the following terms related to chronic pain, marginalized groups and research participation: chronic pain, pain management, chronic pain management, chronic non-cancer pain, marginalised population, marginalised groups, vulnerable populations, marginalised persons, low income, drug users, racialized, ethnic groups, Indigenous peoples, Indigenous, African Americans, homeless, homeless persons, addiction, substance-related disorders, mental health, mental illness, mental disorders, research subjects, patient participation, research participation, patient engagement, framework, lived experiences in research, shared decision making, patient engagement committee, community-based participatory research, health status disparities, participatory action research, people with lived experiences. These keywords were in alignment with our working concept of marginalization as noted above. By including the broad term of chronic pain within the search strategy, we were prepared to receive results that spoke to varying diagnoses, mechanisms, and experiences. Incorporating this heterogeneity of chronic pain reflects our overall interest in the social aspects of chronic pain through the lens of marginalization. We could not assume a similar experience for those who, for example, have attributable causes for their chronic pain as this would negate how the social (i.e., relations, organization, institutions, etc.) is foundational to experience.

The inclusion criteria were primary or secondary research approaches published in English that focused on chronic pain and marginalization. Primary and secondary sources were included because we were interested in conceptualizations and operationalization of marginalization in the scientific literature in general. We excluded studies focused on cancer pain, acute pain, as well as commentaries and abstracts. There were no date limits applied to the search. In total, 926 studies were imported into Covidence® for screening with 355 duplicates removed. This resulted in 571 studies for screening.

Titles and abstracts were independently and collaboratively screened in September 2021 by LC, FW, RL and MS. We identified 83 studies that met the inclusion criteria, of which three were inaccessible. Between December 2021 and January 2022, the lead and senior authors (LC and FW) screened approximately 10% of the studies for relevance. The remaining studies were then divided among four authors (LC, FW, RL and MS) for full-text review, which was followed by data extraction. This screening of the 80 studies resulted in a total of 50 studies included in the scoping review. Reasons for exclusion at full text review included: methodological papers; and, attention to opioid tapering instead of chronic pain (Figure 1). From the 50 included studies, the same four authors then extracted key data elements, including: participants, country, purpose, design, theory used, and findings. Each author reviewed carefully and had taken notes of the literature they were assigned. Then, during a virtual meeting, the authors discussed the data and identified themes. Extensive notes were taken of the meeting and the studies to create an audit trail. The team then organized the data into themes. The lead author (LC) then re-read the entire data set to ensure accuracy.

We conducted an updated search in November 2022 to fill the gap from 2021 onwards. This resulted in 157 additional abstracts to screen in Covidence® with 87 duplicates removed. Upon review of the abstracts, 33 were included for full text screening, and 17 of the full texts were included in the review (Figure 1). Members of the team (LC, FW, MS) met virtually in May 2023 to discuss the new data and identify if any new themes needed to be developed. Extensive notes were taken during the meeting and the studies to create an audit trail. The lead author then compared the themes from the second review with those of the first review. In total, 67 studies were included in the scoping review study.

FIGURE 1 HERE

The preliminary findings were presented to three researchers on the COPE II team (AS, JK, CD) and we drew on their feedback in the scoping review in the consultation phase to enhance the relevance of our results and analysis. Although such forms of consultation are viewed as optional,^13,20^ this is an element of the scoping review that is useful and important^20,21,24,25^ given the multi-disciplinary views they contributed from pain psychology, family medicine and nursing. A final version of the manuscript was then reviewed by all authors. A review protocol was not published for this study.

**Findings**

We endeavoured to present our findings (see Appendix A) in a way that speaks to the expectations of both traditional scoping reviews, and scoping reviews that apply a critical lens. As a result, we divide our findings below into descriptive and critical syntheses. The categories that we developed for the descriptive review include: populations, theory and frameworks, and primary purposes of the literature. Through critical analysis, we developed the following three inter-related themes: 1) varying considerations of social-political and socio-economic contexts; 2) conceptual conflations between sex and gender; and 3) differing approaches to how people living with chronic pain and marginalization were described. We use these themes to highlight what is missing or what requires ongoing work and to offer some suggestions for future research directions.^[[2]](#footnote-3)^

***Descriptive Synthesis***

All of the studies included in the scoping review (n=67) were published between 1999 to 2022, with 46 (68%) published since 2018. Two-thirds of the studies were published in medical and clinical journals (n=44, 66%), followed by a combined category of other journals (encompassing psychology, humanities, and inter/multi-disciplinary) (n=19, 28%), and four (n=4, 6%) graduate dissertations. The top representative journals were the *Journal of Pain* (n=6) and *Pain Medicine* (n=6), followed by the *Journal of General Internal Medicine* (n=3), *PAIN* (n=3), *Pain Management Nursing* (n=2), *Global Qualitative Nursing Research* (n=2), *Disability and Rehabilitation* (n=2), and *International Journal of Environmental Research and Public Health* (n=2). The regional focus for the studies were the United States (n = 45, 67%), Canada (n = 7, 10%), Europe (Denmark, Spain and Sweden, n = 5), Australia and/or New Zealand (n=4), Singapore (n=1), Vietnam (n=1), and Canada and the United States (n=1). Three (n=3) studies were deemed not applicable in this regard. In terms of methods, 26 (39%) studies were qualitative and 30 (45%) were quantitative (which included randomized controlled/clinical trials), four (n=4, 6%) were mixed-methods, and seven (n=7, 10%) were reviews (including literature, narrative, meta-ethnography, and systematic).^16,26-31^ The most frequently cited method of data collection was interviews (n=21, 31%), followed by questionnaires/surveys (n=15, 22%). Most studies (n=49, 73%) focused generally on chronic pain with the remaining focusing on specific chronic pain conditions, including migraine, fibromyalgia, sickle cell disease, diabetic neuropathy, HIV-related pain, irritable bowel syndrome, hip, or knee osteoarthritis, and back, neck or knee pain.

1. *Populations*

The populations included in the studies were: sex workers, people who use drugs, people living with mental illnesses, veterans, migrants and refugees, elderly people, Indigenous Peoples, Black people, and people of colour (i.e., not Black and not Indigenous), women, people living with HIV, and people living with low income. Considering our focus on marginalization and EDI, we narrowed in on the inclusion of women, Indigenous Peoples, Black people, and people of colour to highlight those studies that aimed to confront legacies of racism and sexism and the historical neglect of these populations within chronic pain research. Within the studies included in the scoping review, 14 (21%) exclusively focused on women,^31-44^ 12 (18%) exclusively focused on Black people,^26,28,34,36,37,45-51^ three (n=3) exclusively focused on Indigenous Peoples,^52-54^ and six (n=6) exclusively focused on people of colour.^41,44,55-58^

In the 12 studies that focused on chronic pain among Black people, some of the findings include the influence of environmental, socioeconomic, and racial influences on chronic pain,^28^ lack of empathy and inadequate treatment within clinical spaces,^46^ medical discrimination,^36^ intergenerational empathy,^49^ social and familial characterizations and expectations,^37^ and pain coping.^50^ In the three studies that focused on chronic pain among Indigenous Peoples, emphasis was on the endorsement of traditional health practices within treatment programs^52^ and their placement within broader contexts of colonization,^53^ as well as the development of a culturally responsive online pain management program.^54^ Lastly, within the six studies that focused on chronic pain among people of colour, some of the findings assessed the multiple dimensions of racialized discrimination,^57^ and indicated the importance of culturally relevant online health education interventions,^41^ culturally adapted physiotherapy,^56^ and holistic interventions in addressing pain in light of the role of adversity on pain experiences.^44^

1. *Theories and frameworks*

A large portion of the studies (n = 28, 42%) did not specify a theory or guiding (conceptual) framework. Of those studies that used a framework (n=39, 58%), examples include Black feminist thought and critical arts-based inquiry,^34^ participatory action research (PAR),^54^ Gadamerian philosophical hermeneutics,^35^ two nested hierarchical models (Bronfenbrenner’s bioecological model and Social Ecological Model),^16^ National Institute on Aging (NIA) Health Disparities Research Framework,^30^ Newman’s theory,^47^ sociology of illness experience,^59^ Rhodes’ Risk Environment framework,^60^ Bronfenbrenner’s Process-Person-Context-Time Model,^45^ Neuman’s Systems Model,^61^ life course perspective,^44^ and the biopsychosocial model.^27,29,31,42,62,63^ We noted that the studies that drew on theory offered more fulsome understandings and approaches to marginalization that shed light on the structures underpinning aspects of chronic pain experience among marginalized groups. For example, through the lens of Black feminist thought, Anthym^34(p18)^ powerfully illuminates “the interlocking oppressions of race, gender, and class experienced by Black women” while “center[ing] alternative sources of knowledge, as well as alternative methods of knowledge validation”. Nevertheless, only a minority of studies clearly offered a theoretical view of the processes of marginalization. While some authors (n=12, 18%) operationalized the term marginalization or marginalized, only two (n=2) offered clear definitions.^16,34^

1. *Primary Purposes of the Literature*

We identified three primary purposes for the studies: 18 (27%) focused on the experiences of people living with chronic pain (which can include, but is not limited to, discrimination and stigma); 17 (25%) focused on mental illness and/or substance use among those living with chronic pain; and nine (n=9, 13%) focused on intervention focused research. Combined, these three primary purposes represent 66% (n=44) of the studies included in the scoping review. We describe these below. Note that due to the large number of included studies, we are unable to provide a representative description. Instead, what is presented is a general overview of some of the studies with emphasis placed upon individual studies; review studies did not necessarily capture all the studies included in this scoping review nor analyze the data in a way that is similar to our approach.

**3.1. *Experiences of people living with chronic pain:*** Focusing on the experience of living with chronic pain was the most common motive for the studies. Here, approaches to experience varied with regard to methodology and population. For example, Allen and colleagues^32^ conducted an exploratory qualitative analysis in order to understand the experiences of chronic pain among female survival sex workers in Vancouver’s downtown east side. Through their work, they found that chronic pain may be understood as a symptom of the complexities of marginalization, which include trauma, mental illness, drug use, poverty, and stigma. For Arman et al.,^35^ they aimed to understand women’s experiences of chronic pain through a Gadamerian philosophical hermeneutics approach and caring science and gender perspectives. Through their work, they highlight the social and cultural impacts (including gender norms) upon chronic pain. Booker and colleagues^48^ focused on experiences of osteoarthritis pain among aging African Americans through a qualitative descriptive design. They suggest the conceptual frame of ‘bearing the pain’ to illuminate the process through which older African American adults live with pain, thereby offering insights on personal and cultural ways of self-management that have implications for care practices.

**3.2. *Mental illness and/or substance use:*** The second most common research focus was mental illness and/or substance use among people living with chronic pain. For example, by focusing on experiences of chronic pain among those who use street opioids and/or cocaine, researchers^59^ were able to illustrate the “double stigmatization” faced by those living with chronic pain and labelled as “drug addicts.” This also shed important light on how “the chronic pain experience appeared to be a peripheral experience” in everyday life.^59(p4)^ Another study^64^ focused on hospitalized adults which brought into focus trauma and homelessness, and the barrier that pain presents to stopping drug use. Here, hospitalization and mortality were presented as a motivational factor for change. In addition to the focus on drug/substance use, studies also focused on mental illness (i.e., anxiety and/or depression) or a combination of mental illness and drug use. For example, Poleshuck et al.^43^ asserted through their quantitative work that comorbid pain and depressive symptoms are common among financially disadvantaged women. Naushad and colleagues^65^ aimed to understand the experiences and social consequences of living with depression and chronic pain, and their findings indicate higher levels of self-reported perceived stigma than those living with chronic pain or depression alone. Vogel and colleagues^66^ quantitatively examined the interdependence between chronic pain, substance use and mental illness among individuals experiencing homelessness, suggesting an association between chronic pain and severity of substance use. Through these studies, we learn about the importance of accounting for other health factors, like drug/substance use and mental illness, in understandings and treatments of chronic pain.

**3.3 *Intervention focused research/studies:*** The final key focus of the included studies was health education programs, training programs, and pain management programs. For example, Bruns and colleagues^67^ examined the psychosocial experiences of low-income ethnically diverse people living with pain before and after their participation in an Integrative Pain Management Program. Their findings indicate an increase in resilience and social connections through the uptake of new management strategies and perspectives, highlighting the importance of integrative medicine groups. Pagán-Ortiz and Cortés^41^ examined the acceptance of, and satisfaction with, an online health intervention for Spanish-speaking Latin women living with chronic pain. They paid specific attention to health literacy and empowerment, which highlights its feasibility and impact, and the need for such interventions. Lastly, Perry et al.^54^ outlined their modified PAR framework in the development of a culturally relevant online pain management program with Māori in Aotearoa (New Zealand). Through this combined work, readers are made aware of how and in what ways communities can better understand and manage their chronic pain.

**Findings: Critical Synthesis**

The above descriptive synthesis sheds light on important contributions by researchers in the field by offering a ‘map’ of the chronic pain terrain. In this section, our critical lens draws attention to risks and fissures within this literature.

1. ***Varying considerations of socio-political and socio-economic contexts***

To conduct research in relation to marginalization necessitates acknowledgement of and engagement with the social (i.e., relations, organization, institutions, etc.). For example, one study presented different “systems” or models (such as socio-political) to highlight the different factors that inform clinical conversations on chronic pain,^16^ while another employed a socio-cultural level of analysis.^30^ Studies that accounted for social context allowed them to propose equity-oriented healthcare approaches^10^, underline racialized discrimination^57^ and the role of neighbourhood influences,^28^ highlight connections between pain and exile,^36^ and accentuate the misalignment between biomedical perspectives and the lived realities of women with chronic pain.^33^ Through these studies, which attempt to grapple with the socio-political and/or socio-economic aspects of chronic pain, we learn of the complex historical and contemporary forms of inequity that are embedded within chronic pain experiences and treatments.

In the 67 studies included in this scoping review, 19 (28%) offered little to no engagement with the social aspects of chronic pain; the lack of attention to, and engagement with, social aspects in the broader literature was noted in one study.^30^ For example, exploring the relationship between stressors (a term that focuses on the person) and the quality of life of people living with chronic pain can lead authors to emphasize individual solutions, such as offering time management strategies.^61^ While perhaps a useful downstream tool at the individual level, time management on its own is insufficient to address the upstream forces that sustain processes of marginalization, as would calling for affordable and adequate social supports to assist in the management of pain.

The conceptualization of the socio-political and socio-economic aspects of chronic pain were not clear or consistent within the collected literature. We noted references to marginalization/marginalized, disparity, underrepresented, undertreated, perceived injustice, and vulnerability, yet little to no definitions were provided and many were often used interchangeably. Without conceptual clarity, researchers risked misinterpreting the realities of life in a social world and conflating conceptually distinct terminology. Furthermore, conceptual clarity and consistency ensures researchers stay focused on the broader social issues that inform chronic pain experience and treatment.

There are risks, however, that even with sustained attention to groups who have been marginalized and who face issues such as limited social support, unstable housing, etc., researchers may (unwittingly) suggest individual solutions like acupuncture^68^ or develop interventions that target “exercise, psychological wellbeing, regaining function, emotional wellbeing, sleep hygiene, and stress management”^41(p12)^ that do not necessarily take social barriers into account.^48,54^ While authors reported some modest positive effects of these interventions, it is questionable whether such individualized solutions are sufficient for addressing complex health and social issues which drive marginalization.

1. ***Conceptual conflations between sex and gender***

We noted how gender rather than sex was often not accounted for in most studies, and that it was often not clearly defined. Indeed, only two definitions of gender were provided: gender is “a social structure, interwoven with reproductive processes, identity, and power that differs between cultural and historical contexts”;^35(p773)^ and, gender is a social construct, “refer[ring] to social and cultural expectations, beliefs, and norms” that are “contextualized and recurrently created though social interactions.”^63(p2)^ Without clear definitions of gender, researchers may miss how it is socially constructed, may employ gender descriptively, and/or may conflate it with sex.

As noted by some of the authors included in this scoping review, women face disparities within the field of chronic pain care,^31^ and these can stem from the social construct of gender.^33,35,36^ We anticipated a high percentage of studies that would take into account women’s gendered experiences of chronic pain, but there was limited focus (n=14, 21%) on women exclusively within the studies included in this scoping review. Only six (n=6, 43%) of these 14 studies focusing on chronic pain in women referred to gender in clear distinction from sex,^31,34-37,39^ while three (n=3, 21%) failed to incorporate it at all.^38,43,44^ We found this to be a surprising finding. Many of the chronic pain conditions faced by women, like fibromyalgia for example, are often not effectively managed nor understood which, according to one study, necessitates a “gender-sensitive perspective.”^33^ Attending to gender can allow researchers to better account for those socially constructed aspects of chronic pain that affect women, like (paid and unpaid) work and overperformance, biases, family life and obligations (i.e. caregiving), embodiment, and other struggles and life situations.^33,35^ Doing so may also assist in illuminating processes of marginalization as they pertain to women living with chronic pain. More research is needed that accounts for gender and for women’s gendered experiences of chronic pain.

However, as argued within two studies included in this review^36,37^ it is not enough to simply consider gender. Gender is but one social factor that shapes experiences of chronic pain; it intersects and is intersected by various other social identities.^36^ For example, while Dugan et al.^39^ call attention to the limited engagement with gender in analyses of discrimination and pain, over half of their respondents were white and highly educated, which yields a highly specific and skewed understanding of the role of gender in the lives of a specific group of women. Of the 14 studies focusing exclusively on women, five (n=5, 36%) took into account how gender intersects with racialized identity,^34,36,37,41,44^ with three (n=3, 21%) of these studies focusing on Black women^34,36,37^ and two (n=2, 14%) on Latinx women^41,44^. Four of the studies also either included, or made reference to the need for, a non-binary understanding of gender.^10,60,67,69^

1. ***How people living with chronic pain and marginalization are described***

We took note of differing approaches to how people living with chronic pain and marginalization were described, including diversity in the use of person-first language. Person-first language (person precedes disability, such as “persons living with chronic pain”) was prominent in 37 (55%) studies. Identity-first language (disability precedes person, such as “chronic pain patient”) was prominent in 13 (19%) studies; both were prominent in 17 (25%) studies. We also noted some contentious language. Examples include “impoverished medical populations”,^70^ “impoverished minorities”,^28^ “indigent adults”,^47^ and “vulnerable patients”^16,67,71^ that may situate blame with the person living with pain and perpetuate harmful stereotypes. While the term “participant” was dominant, this sometimes occurred alongside the terms of “subject” and “patient” which can also individualize and medicalize and strip away the identities of people living with pain. There were also references to “drug abuse”^46,47,72,73^ and “substance abuse”^27,50,62,73,74^ which are terms laden with negative connotations, stigma, and individual blame. Other references of concern include resilience, capacity, and empowerment which are individualized notions that may obscure systemic and structural issues that also contribute to chronic pain.

We noted that in many studies researchers often employed broad, sweeping categories—such as “women”, “African Americans”, “Hispanics”, or “American Indian people”—when discussing chronic pain within heterogeneous populations. Of course, focusing on experiences of chronic pain within these populations is needed, yet such categorizations risk sustaining a problematic assumption of a universal experience of living with chronic pain that is void of complex social contexts. Flattened characterizations do not allow researchers to gain adequate insights on the complexities defining chronic pain and marginalization, let alone the diversity of experience within groups.

**Discussion**

The scoping review provides an account of the contributions made by researchers to understanding chronic pain within marginalized settings. It also calls attention to fissures. We draw on these findings to provide some direction on future research on chronic pain and marginalization. Specifically, highlighting the importance of conceptual clarity, including social aspects of experience, and attending to language use as it pertains to chronic pain may contribute to the advancement of more socially just scholarship. Given the relative lack of shared conceptual understandings and definitions of marginalization as a social process, we argue that theoretical approaches grounded in the social sciences—especially with critical leanings—could offer new and innovative ways forward in the field. Indeed, editors from leading pain journals like the *Canadian Journal of Pain* have endorsed the use of “social frameworks for interpretations” in their proclamation to eliminate disparities and support inclusive environments in pain science, scholarship, and publishing.^6(p107)^

It has recently been asserted that we are witnessing an “emergent” generation of pain disparities research that is rooted in justice.^75^ To usher in this generation, one immediate action is to “re-shape our thinking” about chronic pain via new and/or different conceptualizations and re/definitions.^75(p6)^ Recent work undertaken by our team underscores the importance of introducing new conceptualizations, like the concept of chronic struggle, to nuance understandings of largely biological notions of chronic pain when describing the experiences of people with chronic pain who face marginalization.^19^ In order to move towards new and/or different conceptualizations and re/definitions, we must be aware of the existing need for conceptual clarity and careful application, as made evident in this scoping review and by others.^76^ Simply adopting concepts, like marginalization for example, without clear definition can perpetuate conceptual imprecision and/or inaccuracies in practical applications. This in turn can hinder innovative insights and interventions that draw attention to social and structural issues.

Indeed, one of the key findings of this scoping review is the lack of clear definitions. However, even when definitions are provided they may not necessarily call into question the embedded assumptions that underpin them. For example, while the terms vulnerability, resilience, and empowerment are prominent in chronic pain scholarship, and evident within the scoping review, they may direct our focus to the individual which steers attention away from critically analyzing the role of social systems, structures, and relations in people lives. To be clear, when authors incorporate such terms, they are not defining or intending to use them in ways that negatively characterize or impact people living with chronic pain. However, the individualized aspects of terms (that focus on the biological or psychological) may unintentionally facilitate adverse effects or outcomes by perpetuating stigma and/or assumptions of inherent weakness or deficiency.^77,78^ It is in this regard that the use of terms requires care and caution particularly when applied within contexts of marginalization. Based on our findings, the employment of vulnerability and other above-mentioned concepts within chronic pain research that begins in contexts of marginalization may be less useful as they may risk obscuring systemic and structural barriers that inform chronic pain and sustaining individual blame. Bringing in a focus on the social aspects of chronic pain, for example through the concept of marginalization, can be complementary and offer opportunities to further explore the mechanisms that lead to some groups facing greater barriers to care and worse outcomes.

In keeping with our focus on language, person-first language was prominent in over half of the studies included in the scoping review. This is indeed a contentious aspect of communication.^79^ Some argue person-first language may sustain stigma, as it is typically only used with people living with ‘undesirable’ conditions as compared to those without.^80^ By contrast, others assert a need for person-first language to assist with de-stigmatization.^81^ We support the use of person-first language within the chronic pain context, which is also reflected in leading pain journals.^6^ Person-first language supports the calls of people living with pain to be acknowledged as such—as people with specific and unique social experiences.^82^ With recent calls for EDI in pain science, which includes the use of “language that is inclusive and minimizes bias”,^6(p106)^ we urge researchers to be more attentive to how they refer to people living with chronic pain and marginalization.

Our focus on gender highlighted a paucity of studies that accurately engage with the concept, which has been noted by others as a key challenge in pain research.^83^ This is in light of calls for both gender-sensitive research^33^ to account for the influence of gender on women’s experiences of chronic pain,^84-87^ and for greater critical awareness of the “gender paradox of pain”, defined as the “persisting gender biases in health care and their seeming acceptance”.^31(p491)^ Calls to incorporate gender as an analytical lens are also supported by national funding bodies in the health sciences of which 79% of the studies included in this scoping review are based.^88,89^ We support such calls, especially with regard to chronic pain in women. Women are more affected by chronic pain than men^90,91^ yet many of the chronic pain conditions most experienced by women are poorly understood, stigmatized, underfunded, and under-researched.^92,93^ Accounts of illness are shaped by gender discourses, which can sustain skepticism, rejection, and/or disregard.^87,94^ A gendered approach to chronic pain can shed light on the socio-political aspects of this disease, allowing for better understandings and treatments.

However, it is important to approach gender with care. There are risks in preserving a binary gender division,^10^ which can sustain pain disparities among gender diverse people who are at greater risk of discrimination or denial of care within healthcare settings.^95^ It is for this reason that health professions education must attend to the needs and issues of the 2SLBGTQIA+ community.^96,97^ A related risk is the denial of heterogeneity, intersecting identities, and oppressions.^37,98,99^ Accounting for the ways that racialized identity, socio-economic status, geographic location, ability, sexual identity, and other categories intersect with gender would provide nuanced insights on experiences of chronic pain.^100^ Yet, in this scoping review, we noted how intersections of gender and racialized status have yet to be sufficiently addressed in chronic pain scholarship. Racialized populations face inequitable barriers when it comes to pain treatment and management,^101-104^ and illuminating how racialized status intersects with gender is necessary to further advance our understandings of these inequities. However, researchers caution against relying solely on biological restrictions of race that can sustain a separation from the social—namely, racism.^105-108^ As argued by Hood et al.,^109(p922)^ “when we do not examine racism and its effects, troubling assertions of unmeasured biological or genetic reasons for racialized differences in pain outcomes can occur”. This scoping review points to a need for socially grounded scholarship that can account for intersecting identities and multiple and intersecting sources of oppression and inequity. The review also suggests a need for antiracism research practices in chronic pain scholarship,^110^ and other similar research practice recommendations, such as the use of EDI-D frameworks.

**Recommendations for Future Studies on Chronic Pain and Marginalization**

The aim of this scoping review was to map out how studies of chronic pain and marginalization were carried out, how people living with chronic pain who are subjected to marginalization are imagined and discussed, and how marginalization is conceived within the literature. In doing so, we hoped to identify valuable contributions and lingering gaps to assist the field in moving forward. Below, we detail some potential strategies for future research on chronic pain and marginalization. These are intended to complement those strategies already offered by others in the field.^75^ Based on this scoping review, we offer the following inter-related suggestions:

1. Offering clear accounts of socio-political and socio-economic contexts of chronic pain may help to extend understandings beyond individual level clinical explanations, which is pertinent within contexts of marginalization. This broader approach is often found within social science literature and critical scholarship that accounts for the broader social influences that inform people’s lives, like social norms, language use, and socio-political conditions. Including and working with social scientists and critical scholars when conducting chronic pain research could also help to achieve this.^[[3]](#footnote-4)^ Without an account of the social aspects that inform the chronic pain experience, researchers risk rendering people responsible for their experiences by disregarding pre-existing and often long-standing barriers and inequities.
2. Future research could provide clear definitions and explanations of terminology and guiding theoretical or conceptual frameworks. Others have similarly discussed issues regarding terminology in the chronic pain space.^76,112^ Additionally, we note the importance of describing people/participants not in broad homogenous categories but rather in ways that account for intersecting identities, which has also been noted by others.^113^ Addressing these issues offers transparency, reduces conceptual conflation, and avoids unintentionally harmful concepts or biased language. Safety and dignity of people living with chronic pain and marginalization is one of the cornerstones of EDI-D of which language plays a key role.
3. Accounting for the role of gender in research may better prepare researchers to explicate the nuances of chronic pain and marginalization. By gender, we are referring to the social construct that includes socially and culturally ascribed norms, roles, expectations, identity, and expression which—for the purposes of this study—shape experiences of pain (such as how it is recognized and treated).^84,114,115^ It is different from the biologically and physiologically rooted concept of sex, yet it often remains conflated in the literature. Utilizing a gender lens assists in highlighting the inequity, discrimination, and dismissal that, for example, women with chronic pain may face in their daily social and clinical encounters.^85-87^ It can also shed important and much needed light on the unique experiences of chronic pain among gender diverse people. While gender is a defining element of health, it is one of many social aspects defining peoples’ lives. Incorporating an intersectional theoretical lens may be one means to avoid homogeneous discussions of populations, which can flatten understandings of chronic pain, by illuminating how for example gender, racialized status, and socio-economic status influence chronic pain experiences. Other recommendations for future research based on our findings, and those of others,^83^ include the incorporation of clear and accurate definitions of gender and interrogating potentially harmful and exclusionary assumptions of gender.

**Limitations**

We are aware of the limitations of language, and its changing nature. We may have included terms that may not be used by all researchers or communities, or we may have missed terms that are only newly emerging. We also note a risk in our search strategy. By using the guiding concept of marginalization we may have missed research that did not employ this concept or other synonymous concepts. By naming communities as marginalized we acknowledge the risk of applying a term that might not be accepted by all communities or individuals identifying with those communities. Lastly, our inclusion of chronic pain as a search strategy yielded varying diagnoses, mechanisms, and experiences. The incorporation of this term was intended to capture all aspects of chronic pain through the lens of marginalization. However, this may have also influenced findings as certain chronic pain conditions (i.e., arthritis) have greater support within medical frameworks, leading to a significantly different experience than those living with conditions that have less support (i.e., fibromyalgia).^116^ We do not want to assume that social aspects do not play a role within this chronic pain hierarchy and it is for this reason that the broad use of the term chronic pain was applied within the search.

**Conclusion**

Increasing attention is being paid to the role of EDI-D frameworks as it pertains to people who are historically and structurally marginalized and living with chronic pain. Doing so renders visible the diverse social aspects that come to define and shape experiences of chronic pain. However, understandings of what constitutes marginalization and how it is approached within the chronic pain literature varies. This review identified that: (1) greater conceptual clarity and application of marginalization within chronic pain research is required; (2) the inclusion of social aspects are necessary in order to make visible processes of marginalization and its effects on chronic pain; and, (3) there is a need to pay attention to how biases may unintentionally enter research studies through language and generalizing references to heterogenous populations. By highlighting these gaps, future studies may be more alert to the importance of incorporating social aspects of chronic pain. This will assist in explicating the implications of marginalization and the complexity of experience (beyond the individual) as it is located within systems and structures.

**Author Contributions**

FW conceived of the study, obtained funding, and led the study design. LC led the data collection, analysis and drafting of the manuscript. MSt led the search strategy design and executed the literature search. LC, FW, MS and RL participated in the data abstraction and review. All authors participated in study design, analysis and contributed to the manuscript. All authors read and approved the final manuscript.

**Funding**

This work was supported by the Canadian Institutes of Health Research (CIHR) under Grant 409316 held by Fiona Webster.

**Disclosure of Interest**

Laura Connoy has not declared any conflicts of interest. Michelle Solomon has not declared any conflicts of interest. Riana Longo has not declared any conflicts of interest. Abhimanyu Sud has not declared any conflicts of interest. Joel Katz has not declared any conflicts of interest. Craig Dale has not declared any conflicts of interest. Meagan Stanley has not declared any conflicts of interest. Fiona Webster has not declared any conflicts of interest.

REFERENCES

1. US Association for the Study of Pain (USASP). Diversity, Inclusion and Anti-Racism in Pain SIG. 2023. (Accessed January 5, 2024, at <https://www.usasp.org/diversity--inclusion--and-anti-racism-in-pain>).

2. International Association for the Study of Pain (IASP). IASP Interprofessional Pain Curriculum Outline. 2018. (Accessed January 5, 2024, at <https://www.iasp-pain.org/education/curricula/iasp-interprofessional-pain-curriculum-outline/>).

3. Canadian Pain Task Force. An Action Plan for Pain in Canada. Canada: Health Canada; March 2021.

4. Canadian Institutes of Health Research. Equity, diversity and inclusion resources. 2021. (Accessed January 3, 2024, at <https://cihr-irsc.gc.ca/e/52553.html>).

5. Government of Canada. Tri-Agency Statement on Equity, Diversity and Inclusion (EDI). 2022. (Accessed January 3, 2024, at <https://www.nserc-crsng.gc.ca/InterAgency-Interorganismes/EDI-EDI/index_eng.asp>).

6. Palermo TM, Davis KD, Bouhassira D, Hurley RW, Katz JD, Keefe FJ, Schatman M, Turk DC, Yarnitsky D. Promoting inclusion, diversity, and equity in pain science. Canadian Journal of Pain 2023;7(1):1-11.

7. Janevic MR, Mathur VA, Booker SQ, Morais C, Meints SM, Yeager KA, Meghani SH. Making Pain Research More Inclusive: Why and How. The journal of pain 2021;23(5):707-28.

8. Janevic MR, McLaughlin SJ, Heapy AA, Thacker C, Piette JD. Racial and socioeconomic disparities in disabling chronic pain: findings from the health and retirement study. The Journal of Pain 2017;18(12):1459-67.

9. Dahlhamer J, Lucas J, Zelaya C, Nahin R, Mackey S, DeBar L, Kerns R, Von Korff M, Porter L, Helmick C. Prevalence of chronic pain and high-impact chronic pain among adults—United States, 2016. Morbidity and Mortality Weekly Report 2018;67(36):1001.

10. Wallace B, Varcoe C, Holmes C, Moosa-Mitha M, Moor G, Hudspith M, Craig KD. Towards health equity for people experiencing chronic pain and social marginalization. International Journal for Equity in Health 2021;20(1):1-13.

11. Craig K, Holmes C, Hudspith M, Moor G, Moosa-Mitha M, Varcoe C, Wallace B. Pain in persons who are marginalized by social conditions. PAIN 2019;161(2):261-5.

12. Keralis JM. Pain and poverty: disparities by poverty level in the experience of pain-related interference. Pain Medicine 2021;22(7):1532-8.

13. Arksey H, O'Malley L. Scoping studies: towards a methodological framework. International journal of social research methodology 2005;8(1):19-32.

14. Webster F, Bremner S, Oosenbrug E, Durant S, McCartney CJ, Katz J. From opiophobia to overprescribing: A critical scoping review of medical education training for chronic pain. Pain Medicine 2017;18(8):1467-75.

15. Young IM. Justice and the Politics of Difference*.* Princeton University Press; 1990.

16. Emerson AJ, Einhorn L, Groover M, Naze G, Baxter GD. Clinical conversations in the management of chronic musculoskeletal pain in vulnerable patient populations: a meta-ethnography. Disability and Rehabilitation 2023;45(21):3409-34.

17. COPE II: Chronic Pain Ethnography. COPE: AN INSTITUTIONAL ETHNOGRAPHY OF THE SOCIAL ORGANIZATION OF CHRONIC PAIN CARE. at <https://copestudy.ca>).

18. Smith D, ed Institutional Ethnography as Practice*.* Toronto: Rowman & Littlefield Publishers Inc.; 2006.

19. Webster F, Connoy L, Sud A, Rice K, Katz J, Pinto AD, Upshur R, Dale C. Chronic struggle: An institutional ethnography of chronic pain and marginalization. The Journal of Pain 2023;24(3):437-48.

20. Levac D, Colquhoun H, O'Brien KK. Scoping studies: advancing the methodology. Implementation science 2010;5(1):1-9.

21. Pollock D, Davies EL, Peters MD, Tricco AC, Alexander L, McInerney P, Godfrey CM, Khalil H, Munn Z. Undertaking a scoping review: A practical guide for nursing and midwifery students, clinicians, researchers, and academics. Journal of advanced nursing 2021;77(4):2102-13.

22. Tricco AC, Lillie E, Zarin W, O'Brien KK, Colquhoun H, Levac D, Moher D, Peters MD, Horsley T, Weeks L. PRISMA extension for scoping reviews (PRISMA-ScR): checklist and explanation. Annals of internal medicine 2018;169(7):467-73.

23. Kincheloe JL, McLaren P. Rethinking critical theory and qualitative research. Key works in critical pedagogy: Brill; 2011:285-326.

24. Peters MD, Marnie C, Colquhoun H, Garritty CM, Hempel S, Horsley T, Langlois EV, Lillie E, O’Brien KK, Tunçalp Ӧ. Scoping reviews: reinforcing and advancing the methodology and application. Systematic reviews 2021;10(1):1-6.

25. Peters MD, Marnie C, Tricco AC, Pollock D, Munn Z, Alexander L, McInerney P, Godfrey CM, Khalil H. Updated methodological guidance for the conduct of scoping reviews. JBI evidence synthesis 2020;18(10):2119-26.

26. Aroke EN, Joseph PV, Roy A, Overstreet DS, Tollefsbol TO, Vance DE, Goodin BR. Could epigenetics help explain racial disparities in chronic pain? Journal of pain research 2019:701-10.

27. Baria AM, Pangarkar S, Abrams G, Miaskowski C. Adaption of the biopsychosocial model of chronic noncancer pain in veterans. Pain Medicine 2019;20(1):14-27.

28. Maly A, Vallerand AH. Neighborhood, socioeconomic, and racial influence on chronic pain. Pain Management Nursing 2018;19(1):14-22.

29. Miller TR, Halkitis PN, Durvasula R. A biopsychosocial approach to managing HIV-related pain and associated substance abuse in older adults: A review. Ageing International 2019;44(1):74-116.

30. Patel M, Johnson AJ, Booker SQ, Bartley EJ, Palit S, Powell-Roach K, Terry EL, Fullwood D, DeMonte L, Mickle AM. Applying the NIA Health Disparities Research Framework to Identify Needs and Opportunities in Chronic Musculoskeletal Pain Research. The journal of pain 2022;23(1):25-44.

31. Walker N, Beek K, Chen H, Shang J, Stevenson S, Williams K, Herzog H, Ahmed J, Cullen P. The Experiences of Persistent Pain Among Women With a History of Intimate Partner Violence: A Systematic Review. Trauma, Violence, & Abuse 2022;23(2):490-505.

32. Allen C, Murphy A, Kiselbach S, VandenBerg S, Wiebe E. Exploring the experience of chronic pain among female Survival Sex Workers: a qualitative study. BMC family practice 2015;16(1):1-8.

33. Andersson SI, Hovelius B. Illness‐related complaints in women with chronic widespread pain: importance of a contextual approach. Stress and Health: Journal of the International Society for the Investigation of Stress 2005;21(4):235-44.

34. Anthym M. Now you see me: A black feminist autoethnographic poetic polemic of radical reflexivity and critical arts-based inquiry, University of Denver; 2018.

35. Arman M, Gebhardt A, Hök Nordberg J, Andermo S. Women’s lived experiences of chronic pain: faces of gendered suffering. Qualitative health research 2020;30(5):772-82.

36. Campeau K. Adaptive frameworks of chronic pain: daily remakings of pain and care at a Somali refugee women’s health centre. Medical Humanities 2018;44:96-105.

37. Cousin L, Johnson-Mallard V, Booker SQ. “Be Strong My Sista'”: Sentiments of Strength From Black Women With Chronic Pain Living in the Deep South. Advances in Nursing Science 2022;45(2):127-42.

38. Daffin M, Lynch-Milder MK, Gibler RC, Murray C, Green CM, Kashikar-Zuck S. A qualitative study of risk and resilience in young adult women with a history of juvenile-onset fibromyalgia. Pediatric Rheumatology 2021;19:1-9.

39. Dugan SA, Lewis TT, Everson-Rose SA, Jacobs EA, Harlow SD, Janssen I. Chronic discrimination and bodily pain in a multi-ethnic cohort of midlife women in the Study of Women’s Health Across the Nation. Pain 2017;158(9):1656.

40. Kolotylo CJMM. Exploration of the relationships among personal and illness-related factors, migraine headache pain, the chronic pain experience, coping, depressive symptomatology, disability, and quality of life in women with migraine headache: Nursing, The University of Wisconsin-Milwaukee; 1999.

41. Pagán-Ortiz ME, Cortés DE. Feasibility of an online health intervention for Latinas with chronic pain. Rehabilitation Psychology 2021;66(1):10.

42. Peppard SW, Burkard J, Georges J, Dye J. The lived experience of military women with chronic pain: a phenomenological study. Military Medicine 2023;188(5-6):1199-206.

43. Poleshuck EL, Giles DE, Tu X. Pain and depressive symptoms among financially disadvantaged women's health patients. Journal of Women's Health 2006;15(2):182-93.

44. Walker JL, Harrison TC, Hendrickson SG. Life Course Experiences, Pain and Suffering: A Case Study of an Older Mexican American Woman with Mobility Impairment. Hispanic health care international: the official journal of the National Association of Hispanic Nurses 2013;11(2):53.

45. Washington-Walker J, Moore CL, Whittaker TT, Wagner ML. Predictors of Medical and Vocational Rehabilitation Treatment Compliance Among African Americans with Chronic Pain Conditions: An Exploratory Study. Journal of Applied Rehabilitation Counseling 2017;48(4):7-17.

46. Isenberg SR, Maragh-Bass AC, Ridgeway K, Beach MC, Knowlton AR. A qualitative exploration of chronic pain and opioid treatment among HIV patients with drug use disorders. Journal of opioid management 2017;13(1):5.

47. Crawley JA. The experience of chronic pain as described by African American indigent adults attending an urban primary care clinic. Michigan: Nursing, Wayne State University; 2010.

48. Booker SQ, Tripp-Reimer T, Herr KA. “Bearing the Pain”: The experience of aging African Americans with osteoarthritis pain. Global Qualitative Nursing Research 2020;7:2333393620925793.

49. Booker SQ, Cousin L, Buck HG. “Puttin’on”: Expectations versus family responses, the lived experience of older African Americans with chronic pain. Journal of Family Nursing 2019;25(4):533-56.

50. Allen KD, Arbeeva L, Cené CW, Coffman CJ, Grimm KF, Haley E, Keefe FJ, Nagle CT, Oddone EZ, Somers TJ. Pain coping skills training for African Americans with osteoarthritis study: baseline participant characteristics and comparison to prior studies. BMC musculoskeletal disorders 2018;19(1):1-16.

51. Calhoun C, Luo L, Baumann AA, Bauer A, Shen E, McKay V, Hooley C, James A, King AA. Transition for Adolescents and Young Adults With Sickle Cell Disease in a US Midwest Urban Center: A Multilevel Perspective on Barriers, Facilitators, and Future Directions. Journal of Pediatric Hematology/Oncology 2022;44(5):e872-e80.

52. Greensky C, Stapleton MA, Walsh K, Gibbs L, Abrahamson J, Finnie DM, Hathaway JC, Vickers-Douglas KS, Cronin JB, Townsend CO. A qualitative study of traditional healing practices among American Indians with chronic pain. Pain Medicine 2014;15(10):1795-802.

53. Duwe EAG. Surviving and thriving: an integrated critical theory of chronic pain from stories of urban American Indians living with chronic pain, University of Illinois at Urbana-Champaign; 2016.

54. Perry MA, Devan H, Davies C, Hempel D, Ingham T, Jones B, Reid S, Saipe B, Hale L. iSelf-Help: a co-designed, culturally appropriate, online pain management programme in Aotearoa. Research Involvement and Engagement 2022;8(1):1-15.

55. Wee LE, Sin D, Cher WQ, Li ZC, Tsang T, Shibli S, Koh G. “I'm healthy, I don't have pain”-health screening participation and its association with chronic pain in a low socioeconomic status Singaporean population. The Korean Journal of Pain 2017;30(1):34.

56. Brady B, Veljanova I, Schabrun S, Chipchase L. Integrating culturally informed approaches into physiotherapy assessment and treatment of chronic pain: a pilot randomised controlled trial. BMJ open 2018;8(7):e021999.

57. Walsh KT, Boring BL, Nanavaty N, Guzman H, Mathur VA. Sociocultural context and pre-clinical pain facilitation: multiple dimensions of racialized discrimination experienced by latinx Americans are associated with enhanced temporal summation of pain. The Journal of Pain 2022;23(11):1885-93.

58. Nguyen AT, Nguyen THT, Nguyen TTH, Nguyen HTT, Nguyen TX, Nguyen TN, Nguyen AL, Vu LG, Do HT, Doan LP. Chronic pain and associated factors related to depression among older patients in Hanoi, Vietnam. International journal of environmental research and public health 2021;18(17):9192.

59. Dassieu L, Kaboré J-L, Choinière M, Arruda N, Roy É. Painful lives: Chronic pain experience among people who use illicit drugs in Montreal (Canada). Social Science & Medicine 2020;246:112734.

60. Voon P, Greer AM, Amlani A, Newman C, Burmeister C, Buxton JA. Pain as a risk factor for substance use: a qualitative study of people who use drugs in British Columbia, Canada. Harm reduction journal 2018;15(1):1-9.

61. Gerstle DS, All AC, Wallace DC. Quality of life and chronic nonmalignant pain. Pain Management Nursing 2001;2(3):98-109.

62. Choi NG, Snow AL, Kunik ME. Pain severity, interference, and prescription analgesic use among depressed, low-income homebound older adults. Aging & Mental Health 2016;20(8):804-13.

63. Bureychak T, Faresjö Å, Sjödahl J, Norlin AK, Walter S. Symptoms and health experience in irritable bowel syndrome with focus on men. Neurogastroenterology & Motility 2022;34(11):e14430.

64. Velez CM, Nicolaidis C, Korthuis PT, Englander H. “It’s been an experience, a life learning experience”: a qualitative study of hospitalized patients with substance use disorders. Journal of general internal medicine 2017;32(3):296-303.

65. Naushad N, Dunn LB, Muñoz RF, Leykin Y. Depression increases subjective stigma of chronic pain. Journal of affective disorders 2018;229:456-62.

66. Vogel M, Choi F, Westenberg JN, Cabanis M, Nikoo N, Nikoo M, Hwang SW, Somers J, Schütz CG, Krausz M. Chronic pain among individuals experiencing homelessness and its interdependence with opioid and other substance use and mental illness. International Journal of Environmental Research and Public Health 2022;19(1):5.

67. Bruns EB, Befus D, Wismer B, Knight K, Adler SR, Leonoudakis-Watts K, Thompson-Lastad A, Chao MT. Vulnerable patients' psychosocial experiences in a group-based, integrative pain management program. The Journal of Alternative and Complementary Medicine 2019;25(7):719-26.

68. Liu R, Santana T, Schillinger D, Hecht FM, Chao MT. “It Gave Me Hope” experiences of diverse safety net patients in a group acupuncture intervention for painful diabetic neuropathy. Health Equity 2020;4(1):225-31.

69. Kattari SK, Beltrán R. “The pain is real”: A [modified] photovoice exploration of disability, chronic pain, and chronic illness (in) visibility. Qualitative Social Work 2021:14733250211010902.

70. Phifer J, Skelton K, Weiss T, Schwartz AC, Wingo A, Gillespie CF, Sands LA, Sayyar S, Bradley B, Jovanovic T. Pain symptomatology and pain medication use in civilian PTSD. PAIN® 2011;152(10):2233-40.

71. Chao MT, Hurstak E, Leonoudakis-Watts K, Sidders F, Pace J, Hammer H, Wismer B. Patient-reported outcomes of an integrative pain management program implemented in a primary care safety net clinic: a quasi-experimental study. Journal of General Internal Medicine 2019;34(7):1105-7.

72. Turner BJ, Liang Y, Simmonds MJ, Rodriguez N, Bobadilla R, Yin Z. Randomized trial of chronic pain self-management program in the community or clinic for low-income primary care patients. Journal of general internal medicine 2018;33(5):668-77.

73. Marie BS. Coexisting addiction and pain in people receiving methadone for addiction. Western journal of nursing research 2014;36(4):534-51.

74. Sturycz CA, Hellman N, Payne MF, Kuhn BL, Hahn B, Lannon EW, Palit S, Güereca YM, Toledo TA, Shadlow JO. Race/ethnicity does not moderate the relationship between adverse life experiences and temporal summation of the nociceptive flexion reflex and pain: results from the Oklahoma Study of Native American Pain Risk. The Journal of Pain 2019;20(8):941-55.

75. Booker SQ, Baker TA, Esiaka D, Minahan JA, Engel IJ, Banerjee K, Poitevien M. A historical review of pain disparities research: Advancing toward health equity and empowerment. Nursing Outlook 2023;71(3):101965.

76. MacGregor C, Walumbe J. We need to develop our approach to socially constructed concepts including socioeconomic factors, power, ethnicity and racism in pain care and research. Pain and Rehabilitation-the Journal of Physiotherapy Pain Association 2021;2021(51):1-4.

77. Virokannas E, Liuski S, Kuronen M. The contested concept of vulnerability–a literature review: Vulnerability-käsitteen kiistanalaiset merkitykset–systemaattinen kirjallisuuskatsaus. European Journal of Social Work 2020;23(2):327-39.

78. Katz AS, Hardy B-J, Firestone M, Lofters A, Morton-Ninomiya ME. Vagueness, power and public health: Use of ‘vulnerable ‘in public health literature. Critical Public Health 2020;30(5):601-11.

79. Dunn DS, Andrews EE. Person-first and identity-first language: Developing psychologists’ cultural competence using disability language. American Psychologist 2015;70(3):255.

80. Gernsbacher MA. Editorial perspective: The use of person‐first language in scholarly writing may accentuate stigma. Vol 58: Wiley Online Library; 2017:859-61.

81. Baker EA, Hamilton M, Culpepper D, McCune G, Silone G. The effect of person-first language on attitudes toward people with addiction. Journal of Addictions & Offender Counseling 2022;43(1):38-49.

82. Richards D. The patient as person: an update. British Journal of Sports Medicine 2020;54.

83. Keogh E, Boerner KE. Challenges with embedding an integrated sex and gender perspective into pain research: Recommendations and opportunities. Brain, Behavior, and Immunity 2023.

84. Bendelow G. Pain perceptions, emotions and gender. Sociology of Health & Illness 1993;15(3):273-94.

85. Werner A, Steihaug S, Malterud K. Encountering the continuing challenges for women with chronic pain: recovery through recognition. Qualitative Health Research 2003;13(4):491-509.

86. Werner A, Isaksen LW, Malterud K. ‘I am not the kind of woman who complains of everything’: Illness stories on self and shame in women with chronic pain. Social Science & Medicine 2004;59:1035-45.

87. Werner A, Malterud K. It is hard work behaving as a credible patient: encounters between women with chronic pain and their doctors. Social science & medicine 2003;57(8):1409-19.

88. CIHR. Gender-Based Analysis Plus (GBA+) at CIHR. 2022. (Accessed June 15, 2023, at <https://cihr-irsc.gc.ca/e/50968.html>).

89. National Institutes of Health. What are Sex & Gender? And why fo they matter in health research? (Accessed Nov. 6, 2023, at <https://orwh.od.nih.gov/sex-gender>).

90. Fillingim RB. Sex, gender, and pain: women and men really are different. Current review of pain 2000;4(1):24-30.

91. Rustøen T, Wahl AK, Hanestad BR, Lerdal A, Paul S, Miaskowski C. Gender differences in chronic pain—findings from a population-based study of Norwegian adults. Pain Management Nursing 2004;5(3):105-17.

92. Quintner J. Why are women with fibromyalgia so stigmatized? Pain Medicine 2020;21:882-8.

93. Hoffmann DE, Fillingim RB, Veasley C. The Woman Who Cried Pain: Do Sex-Based Disparities Still Exist in the Experience and Treatment of Pain? Journal of Law, Medicine & Ethics 2022;50(3):519-41.

94. Hoffmann DE, Tarzian AJ. The girl who cried pain: a bias against women in the treatment of pain. Journal of Law, Medicine & Ethics 2001;29(1):13-27.

95. Shires DA, Jaffee K. Factors Associated with Health Care Discrimination Experiences among a National Sample of Female-to-Male Transgender Individuals. Health & Social Work 2015;40(2):134-41.

96. Obedin-Maliver J, Goldsmith ES, Stewart L, White W, Tran E, Brenman S, Wells M, Fetterman DM, Garcia G, Lunn MR. Lesbian, gay, bisexual, and transgender–related content in undergraduate medical education. Jama 2011;306(9):971-7.

97. Abd-Elsayed A, Heyer AM, Schatman ME. Disparities in the Treatment of the LGBTQ Population in Chronic Pain Management. Journal of Pain Research 2021;14(null):3623-5.

98. Bacchi CL. The politics of affirmative action:'Women', equality and category politics*.* Sage; 1996.

99. Pryma J. “Even my sister says I'm acting like a crazy to get a check”: Race, gender, and moral boundary-work in women's claims of disabling chronic pain. Social Science & Medicine 2017;181:66-73.

100. Quiton R, Leibel D, Boyd E, Waldstein S, Evans M, Zonderman A. Sociodemographic patterns of pain in an urban community sample: an examination of intersectional effects of sex, race, age, and poverty status. PAIN 2020;161:1044-51.

101. Akinlade O. Taking black pain seriously. New England Journal of Medicine 2020;383(10):e68.

102. Amen TB, Dee EC, Arega MA, Butler SS, Lee G, Shannon EM, Chen AF. Racial and Ethnic Disparities in Access to Culturally Competent Care in Patients with Joint Pain in the United States. Journal of General Internal Medicine 2021:1-4.

103. Ghoshal M, Shapiro H, Todd K, Schatman ME. Chronic noncancer pain management and systemic racism: time to move toward equal care standards. Journal of Pain Research 2020;13:2825.

104. Hoffman KM, Trawalter S, Axt JR, Oliver MN. Racial bias in pain assessment and treatment recommendations, and false beliefs about biological differences between blacks and whites. Proceedings of the National Academy of Sciences 2016;113(16):4296-301.

105. Hamilton CV, Ture K. Black power: Politics of liberation in America*.* Vintage; 1992.

106. Feagin JR. Racist America: Roots, current realities, and future reparations*.* Routledge; 2014.

107. Bonilla-Silva E. Rethinking racism: Toward a structural interpretation. American sociological review 1997:465-80.

108. Morais CA, Aroke EN, Letzen JE, Campbell CM, Hood AM, Janevic MR, Mathur VA, Merriwether EN, Goodin BR, Booker SQ. Confronting racism in pain research: A call to action. The journal of pain 2022;23(6):878-92.

109. Hood AM, Booker SQ, Morais CA, Goodin BR, Letzen JE, Campbell LC, Merriwether EN, Aroke EN, Campbell CM, Mathur VA, et al. Confronting racism in all forms of pain research: A shared commitment for engagement, diversity, and dissemination. The Journal of Pain 2022;23(6):913-28.

110. Letzen JE, Mathur VA, Janevic MR, Burton MD, Hood AM, Morais CA, Booker SQ, Campbell CM, Aroke EN, Goodin BR. Confronting racism in all forms of pain research: Reframing study designs. The journal of pain 2022;23(6):893-912.

111. Canadian Institutes of Health Research (CIHR). Detailed information. 2023. (Accessed January 5, 2024, at <https://webapps.cihr-irsc.gc.ca/decisions/p/project_details.html?applId=485102&lang=en>).

112. Meghani SH, Gallagher RM. Disparity vs inequity: toward reconceptualization of pain treatment disparities. Pain Medicine 2008;9(5):613-23.

113. Macgregor C, Walumbe J, Tulle E, Seenan C, Blane DN. Intersectionality as a theoretical framework for researching health inequities in chronic pain. British Journal of Pain 2023:20494637231188583.

114. Bendelow G. Pain and Gender*.* Pearson Education; 2000.

115. Bendelow GA, Williams SJ. Natural for women, abnormal for men: Beliefs about pain and gender. In: Nettleton S, Watson J, eds. The body in everyday life. London and New York: Routledge; 1998:199-217.

116. Grue J, Johannessen LE, Rasmussen EF. Prestige rankings of chronic diseases and disabilities. A survey among professionals in the disability field. Social Science & Medicine 2015;124:180-6.

1. Our original question was “What studies have been conducted that engage with or focus on people who live with chronic pain and are subjected to processes of marginalization?”. We found this question caused some confusion among team members and so removed reference to “engage” for clarity. For transparency, we included “engage with” in the original research question to refer broadly to researchers who are engaging with the topic of marginalized groups. The broad scope of this term warranted slight revisions, which were accepted by the Teaching and Learning Librarian (MSt) who assisted with the search strategy and identification of databases. [↑](#footnote-ref-2)
2. These themes are not overly representative; not every reading can be included due to the large body of readings included in this scoping review. [↑](#footnote-ref-3)
3. For example, our COPE II team has been recognized by the Canadian Institutes of Health Science (CIHR) for their ability to address research challenges, like the social aspects of chronic pain, through an interdisciplinary team makeup which blends psychology, medicine, nursing, anthropology, and sociology.^114^ [↑](#footnote-ref-4)
