# Supplementary material for: Attending to Marginalization in The Chronic Pain Literature: A Scoping Review
Source: Can J Pain. 2024 Mar 28;8(2):2335500. doi: 10.1080/24740527.2024.2335500 (PMC11146439; doi:10.1080/24740527.2024.2335500)
Supplement: Supplemental Material [file UCJP_A_2335500_SM2789.docx]

| Reference Number | Reference | Overall purpose or aim of the study | Regional Focus | Journal Type | Methodology | Chronic Pain Focus | Clear theoretical/ conceptual framework | Definition of marginalization | Definition of gender | Clear and thorough engagement with social aspects of chronic pain | Exclusive focus on Indigenous Peoples, Black People, and People of Colour | Exclusive focus on women |
| --- | --- | --- | --- | --- | --- | --- | --- | --- | --- | --- | --- | --- |
| 50 | 1. Allen, K. D., Arbeeva, L., Cené, C. W., Coffman, C. J., Grimm, K. F., Haley, E., ... & Campbell, L. C. (2018). Pain coping skills training for African Americans with osteoarthritis study: baseline participant characteristics and comparison to prior studies. *BMC Musculoskeletal Disorders*, 19(1), 1-16. | Compare characteristics of trial participants with prior  studies of training programs | United States | Medical/ clinical | Quantitative | Specific |  |  |  |  |  |  |
| 32 | 2. Allen, C., Murphy, A., Kiselbach, S., VandenBerg, S., & Wiebe, E. (2015). Exploring the experience of chronic pain among female Survival Sex Workers: a qualitative study. *BMC Family Practice*, 16(1), 1-8. | Understand chronic pain experiences among female survival sex workers in Vancouver’s downtown east side | Canada | Medical/ clinical | Qualitative | General |  |  |  | x | x | x |
| 33 | 3. Andersson, S. I., & Hovelius, B. (2005). Illness‐related complaints in women with chronic widespread pain: importance of a contextual approach. *Stress and Health: Journal of the International Society for the Investigation of Stress*, 21(4), 235-244. | Explain experiences of women with chronic pain who are on sick leave within a contextual framework as defined by the participants | Sweden | Other | Quantitative | General | x |  |  | x |  | x |
| 34 | 4. Anthym, M. (2018). Now you see me: A black feminist autoethnographic poetic polemic of radical reflexivity and critical arts-based inquiry (Doctoral dissertation, University of Denver). | Reveal subjugated truths through the development of a reflexive praxis, an illustration of poetry as a method of inquiry, and a demonstration of lived experience as knowledge | United States | Dissertation | Qualitative | General | x | x |  | x | x | x |
| 35 | 5. Arman, M., Gebhardt, A., Hök Nordberg, J., & Andermo, S. (2020). Women’s lived experiences of chronic pain: faces of gendered suffering. *Qualitative Health Research*, 30(5), 772-782. | Understand women’s lived experiences of chronic pain through a gender perspective | Sweden | Other | Qualitative | General | x |  | x | x |  | x |
|  | 6. Aroke, E. N., Jackson, P., Overstreet, D. S., Penn, T. M., Rumble, D. D., Kehrer, C. V., ... & Goodin, B. R. (2020). Race, social status, and depressive symptoms: a moderated mediation analysis of chronic low back pain interference and severity. *The* *Clinical Journal of Pain*, 36(9), 658-666. | Investigate whether race moderates the relationship between perceived social status and chronic low back pain outcomes, including via depressive symptoms | United States | Medical/ clinical | Quantitative | Specific | x |  |  | x |  |  |
| 26 | 7. Aroke, E. N., Joseph, P. V., Roy, A., Overstreet, D. S., Tollefsbol, T. O., Vance, D. E., & Goodin, B. R. (2019). Could epigenetics help explain racial disparities in chronic pain?. *Journal of Pain Research*, 12, 701. | Explore epigenetics as a mechanism to explain racial differences in chronic pain | United States | Medical/ clinical | Narrative review | Specific | x |  |  | x | x |  |
| 27 | 8. Baria et al. (2019) Adaption of the Biopsychosocial Model of Chronic Noncancer Pain in Veterans. *Pain Medicine*, 20: 14–27. | Adapt Gatchel and colleagues’ review of the bio-psychosocial model of chronic pain to veterans and summarize research findings that support the revised model | United States | Medical/ clinical | Unspecified review | General | x |  |  | x |  |  |
|  | 9. Bean, D. J., Dryland, A., Rashid, U., & Tuck, N. L. (2022). The determinants and effects of chronic pain stigma: A mixed methods study and the development of a model. *The Journal of Pain*, *23*(10), 1749-1764 | Investigate factors that contribute to chronic pain stigma and explore stigma experiences of people with chronic pain | New Zealand | Medical/ clinical | Mixed method | General | x |  |  | x |  |  |
|  | 10. Bernstein et al (2020) Identifying predictors of recommendations for and participation in multimodal nonpharmacological treatments for chronic pain using patient-reported outcomes and electronic medical records. *Pain Medicine*, doi: 10.1093/pm/pnaa203 | Identify predictors of participation in non-pharmacological treatments for chronic pain | United States | Medical/ clinical | Quantitative | General |  |  |  |  |  |  |
| 49 | 11. Booker et al. (2019) “Puttin’ on”: Expectations Versus Family Responses, the Lived Experience of Older African Americans with Chronic Pain. *Journal of Family Nursing*, 25(4), 533-556 | Explore the expectations of familial support among older African American adults living with osteoarthritis and their perceptions of family member support | United States | Medical/ clinical | Qualitative | Specific | x |  |  | x | x |  |
| 48 | 12. Booker et al (2020) “Bearing the Pain”: The Experience of Aging African Americans With Osteoarthritis Pain. *Global Qualitative Nursing Research*, 7, 1–12 | Extend understandings of living with osteoarthritis pain among older African Americans | United States | Medical/ clinical | Qualitative | Specific | x |  |  | x | x |  |
| 56 | 13. Brady B, Veljanova I, Schabrun S, et al. (2018) Integrating culturally informed approaches into physiotherapy assessment and treatment of chronic pain: a pilot randomised controlled trial. *BMJ Open* 2018;0:e021999. | Evaluate feasibility of and patient engagement with a culturally adapted pain management approach | Australia | Medical/ clinical | Quantitative | General |  |  |  | x | x |  |
| 67 | 14. Bruns et al. (2019) Vulnerable Patients’ Psychosocial Experiences in a Group-Based, Integrative Pain Management Program. *The Journal of Alternative and Complementary Medicine*, 25(7), 719-726 | Examine the experiences of low-income, ethnically diverse people living with chronic pain before and after a pain program through a psycho-social framework | United States | Medical/ clinical | Qualitative | General | x |  |  | x |  |  |
| 63 | 15. Bureychak, T., Faresjö, Å., Sjödahl, J., Norlin, A. K., & Walter, S. (2022). Symptoms and health experience in irritable bowel syndrome with focus on men. *Neurogastroenterology & Motility*, *34*(11), e14430. | Analyze experiences of people living with  and without irritable bowel syndrome through a biopsychosocial perspective | Sweden | Medical/ clinical | Quantitative | Specific | x |  | x | x |  |  |
| 51 | 16. Calhoun, C., Luo, L., Baumann, A. A., Bauer, A., Shen, E., McKay, V., ... & King, A. A. (2022). Transition for Adolescents and Young Adults With Sickle Cell Disease in a US Midwest Urban Center: A Multilevel Perspective on Barriers, Facilitators, and Future Directions. *Journal of Pediatric Hematology/Oncology*, *44*(5), e872-e880. | Identify and understand barriers and facilitators of transitioning care for adolescents and young adults with sickle cell disease | United States | Medical/ clinical | Qualitative | Specific | x |  |  | x | x |  |
| 36 | 17. Campeau, K. (2018). Adaptive frameworks of chronic pain: daily remarkings of pain and care at a Somalie refugee women’s health centre. *Medical Humanities*, 44(2), 96-105. | Understand  Somali women’s use of healthcare networks and how they may or may not benefit from medical resources | United States | Other | Qualitative | General | x |  |  | x | x | x |
| 71 | 18. Chao, M.T., Hurstak, E., Leonoudakis-Watts, K., Sidders, F., Pace, J., Hammer, H., & Wismer, B. (2019). Patient-Reported Outcomes of an Integrative Pain Management Program Implemented in a Primary Care Safety Net Clinic: a Quasi-experimental Study. *Journal of General Internal Medicine, 34*(7*), 1105-1107.* | Evaluate the impact of a chronic pain management program among patients at a  clinic | United States | Medical/ clinical | Quantitative | General |  |  |  |  |  |  |
| 62 | 19. Choi, N.G., Snow, A.L., & Kunik, M.E. (2016). Pain severity, interference, and prescription analgesic use among depressed, low-income homebound older adults. *Aging & Mental Health*, 20(8), 804-813. | Examine pain frequency, intensity, and interference; the relationship between pain and depression; and association between use of prescription analgesic and antidepressant and anxiolytic medications among these older adults. | United States | Other | Quantitative | General | x |  |  |  |  |  |
| 37 | 20. Cousin, L., Johnson-Mallard, V., & Booker, S. Q. (2022). “Be Strong My Sista'”: Sentiments of Strength From Black Women With Chronic Pain Living in the Deep South. *Advances in Nursing Science*, 45(2), 127-142. | Identify ways in which older Black women display strength while living with chronic osteoarthritis pain | United States | Medical/ clinical | Qualitative | Specific | x |  |  | x | x | x |
| 47 | 21. Crawley, J. (2010) The experience of chronic pain as described by African American indigent adults attending an urban primary care clinic. (Publication No.3398000) [Doctoral dissertation, Wayne State University]. ProQuest Dissertations Publishing. | Examine chronic pain experiences among low income African American adults at an urban clinic | United States | Dissertation | Qualitative | General | x |  |  | x | x |  |
| 38 | 22. Daffin, M., Lynch-Milder, M. K., Gibler, R. C., Murray, C., Green, C. M., & Kashikar-Zuck, S. (2021). A qualitative study of risk and resilience in young adult women with a history of juvenile-onset fibromyalgia. *Pediatric Rheumatology*, *19*, 1-9. | Identify themes of risk and resilience for outcomes among young adults with juvenile onset fibromyalgia in childhood | United States | Medical/ clinical | Qualitative | Specific |  |  |  | x |  | x |
|  | 23. Dassieu, L., Kaboré, J. L., Choinière, M., Arruda, N., & Roy, É. (2019). Understanding the link between substance use and chronic pain: A qualitative study among people who use illicit drugs in Montreal, Canada. *Drug and Alcohol Dependence*, 202, 50-55. | Improve knowledge of the substance use habits of people who use drugs and who live with chronic pain, and assess the role chronic pain plays in substance use patterns | Canada | Other | Qualitative | General |  |  |  | x |  |  |
| 59 | 24. Dassieu, L., Kabore, J.L., Choinière, M., Arruda, N., & Roy, E. (2020). Painful lives: Chronic pain experience among people who use illicit drugs in Montreal (Canada). *Social Science & Medicine*, 246, 112734-112734. | Understand how experiences of illicit drug use shapes chronic pain experiences | Canada | Other | Qualitative | General | x |  |  | x |  |  |
| 39 | 25. Dugan, S.A., Lewis, T.T., Everson-Rose, S.A., Jacobs, E.A., Harlow, S.D., & Janssen, I. (2017). Chronic discrimination and bodily pain in a multiethnic cohort of midlife women in the Study of Women's Health Across the Nation. *PAIN*, 158(9),1656-1665. | Understand the relationship between discrimination and pain in midlife women | United States | Medical/ clinical | Quantitative | General |  |  |  | x |  | x |
| 53 | 26. Duwe, E.A.G. (2016). Surviving and Thriving: An Integrated Critical Theory of Chronic Pain from Stories of Urban American Indians Living with Chronic Pain. (Publication No.10609649) [Doctoral dissertation, University of Illinois at Urbana Champaign]. ProQuest Dissertations Publishing. | Discern the relationship between chronic pain and colonization and develop a critical theory of chronic pain. | United States | Dissertation | Mixed method | General | x |  |  | x | x |  |
| 16 | 27. Emerson, A. J., Einhorn, L., Groover, M., Naze, G., & Baxter, G. D. (2022). Clinical conversations in the management of chronic musculoskeletal pain in vulnerable patient populations: a meta-ethnography. *Disability and Rehabilitation*, 1-26. | Synthesize research on provider/patient perceptions of  clinical conversations centered on chronic musculoskeletal pain in vulnerable adult populations | Not applicable | Medical/ clinical | Meta-ethnography (Review) | General | x | x |  | x |  |  |
| 61 | 28. Gerstle, D.S., All, A.C., & Wallace, D. C. (2001). Quality of life and chronic nonmalignant pain. *Pain Management Nursing, 2*(3), 98-109. | Explore the impact of stressors on  quality of life among adults living with chronic pain | United States | Medical/ clinical | Quantitative | General | x |  |  |  |  |  |
|  | 29. Grauslund, A.M.H., Michelsen, J.S., & Esbensen, B.A. (2021). Everyday life with chronic back pain: a qualitative study among Turkish immigrants in Denmark. *Disability and Rehabilitation, 43*(8), 1162-1170. | Understand how Turkish immigrants experience back pain | Denmark | Medical/ clinical | Qualitative | Specific | x |  |  | x |  |  |
| 52 | 30. Greensky, C., Stapleton, M.A., Walsh, K., Gibbs, L., Abrahamson, J., Finnie, D.M., Hathaway, J.C., Vickners-Douglas, K.S., Cronin, J.B., Townsend, C.O., & Hooten, W.M. (2014). A qualitative study of traditional healing practices among American Indians with chronic pain. *Pain Medicine, 15*(10), 1795-1802. | Understand the use of traditional healing practices among adult American Indians living with chronic pain | United States | Medical/ clinical | Qualitative | General |  |  |  | x | x |  |
|  | 31. Hooten, W.M., Knight-Brown, M., Townsend, C.O., & Laures, H.J. (2012). Clinical Outcomes of Multidisciplinary Pain Rehabilitation Among African American Compared with Caucasian Patients with Chronic Pain. *Pain Medicine, 13*(11), 1499-1508. | Determine if outcomes of multi-disciplinary pain rehabilitation were different for African Americans compared with Caucasians | United States | Medical/ clinical | Quantitative | General |  |  |  |  |  |  |
| 46 | 32. Isenberg, S.R., Maragh-Bass, A.C., Ridgeway, K., Beach, M.C., & Knowlton, A.R. (2017). A qualitative exploration of chronic pain and opioid treatment among HIV patients with drug use disorders. *Journal of Opioid Management, 13*(1), 5-16. | Explore experiences with pain management regarding clinical access to and use of prescription opioids among people living with HIV and who use drugs | United States | Medical/ clinical | Qualitative | Specific |  |  |  |  | x |  |
| 8 | 33. Janevic, M.R., McLaughlin, S.J., Heapy, A.A., Thacker, C., & Piette, J.D. (2017). Racial and Socioeconomic Disparities in Disabling Chronic Pain: Findings From the Health and Retirement Study. The *Journal of Pain*, 18(12):1459-1467. | Estimate the population prevalence of  high-impact chronic pain among older adults and whether the prevalence and pain intensity vary across groups defined according to race/ethnicity, socioeconomic status, gender and age | United States | Medical/ clinical | Quantitative | General |  |  |  | x |  |  |
| 69 | 34. Kattari, S.K., & Beltrán, R. (2021). “The pain is real”: A [modified] photovoice exploration of disability, chronic pain, and chronic illness (in)visibility. *Qualitative Social Work, 0*(0), 1-19. | Explore experiences of living with non-apparent disabilities, chronic pain and/or chronic illness | United States | Other | Qualitative | General | x |  |  | x |  |  |
|  | 35. Katz, J.N., Lyons, N., Wolff, L.S., Silverman, J., Emrani, P., Holt, H.L., Corbett, K.L., Escalante, A., & Losiina, E. (2011). Medical decision-making among Hispanics and non-Hispanic Whites with chronic back and knee pain: A qualitative study. *BMC Musculoskeletal Disorders*, 12(1), 78 | Understand differences in medical decision making among Hispanics and non-Hispanic Whites according to socioeconomic indicators | United States | Medical/ clinical | Qualitative | Specific | x |  |  |  |  |  |
| 40 | 36. Kolotylo, C.J.M. (1999). Exploration of the relationships among personal and illness-related factors, migraine headache pain, the chronic pain experience, coping, depressive symptomatology, disability, and quality of life in women with migraine headache. (Publication No. 9926921) [Doctoral dissertation The University of Wisconsin-Milwaukee]. ProQuest Dissertations Publishing. | Determine relationships between personal and illness related factors among women living with migraine headache | United States | Dissertation | Quantitative | Specific | x |  |  | x |  | x |
|  | 37. Lewis, G. N., & Upsdell, A. (2018). Ethnic disparities in attendance at New Zealand’s chronic pain services. *The New Zealand Medical Journal, 131*(1472), 21–28. | Determine if access to chronic pain services in New Zealand is equitable and if differences exist in baseline presenting characteristics of patients | New Zealand | Medical/ clinical | Quantitative | General |  |  |  | x |  |  |
| 68 | 38. Liu, R., Santana, T., Schillinger, D., Hecht, F. M., & Chao, M. T. (2020). “It Gave Me Hope” Experiences of Diverse Safety Net Patients in a Group Acupuncture Intervention for Painful Diabetic Neuropathy. *Health Equity, 4*(1), 225–231. | Explore experiences of diabetic neuropathy and group acupuncture intervention in low-income patients | United States | Other | Quantitative | Specific |  |  |  | x |  |  |
| 28 | 39. Maly, A., & Vallerand, A. H. (2018). Neighborhood, Socioeconomic, and Racial Influence on Chronic Pain. *Pain Management Nursing: Official Journal of the American Society of Pain Management Nurses, 19*(1), 14–22. | Highlight environmental, socioeconomic, and racial influences on chronic pain among African Americans in low-income urban neighbourhoods | United States | Medical/ clinical | Unspecified review | General |  |  |  | x | x |  |
| 29 | 40. Miller, T. R., Halkitis, P. N., & Durvasula, R. (2019). A biopsychosocial approach to managing HIV-related pain and associated substance abuse in older adults: A review. *Ageing International, 44*(1), 74-116. | Examine associations between substance use, mental health, psychosocial issues, and chronic pain through a bio-psychosocial framework | Not applicable | Other | Literature review | Specific | x |  |  | x |  |  |
|  | 41. Miguel-Cruz, A. M., Brintnell, S., Roxburgh, M., Salamanca, J. G., & Liu, L. (2021). Psychosocial risk factors and their impact on the performance of everyday activities in Canadian veterans. *Journal of Military and Veterans Health*, *29*(4), 28-41 | Identify correlations of psychosocial risk factors with symptom profile factors and perceived impact of health conditions on daily activities | Canada | Medical/ clinical | Quantitative | General | x |  |  |  |  |  |
|  | 42. Mittinty, M. M., Elliott, J. M., Hunter, D. J., Nicholas, M. K., March, L. M., & Mittinty, M. N. (2022). Explaining the gap in the experience of depression among arthritis patients. *Clinical Rheumatology*, 1-7. | Explain factors contributing to the gap in depression between employed people with and without paid sick leave who live with arthritis | United States | Medical/ clinical | Quantitative | Specific |  |  |  | x |  |  |
| 65 | 43. Naushad, N., Dunn, L. B., Muñoz, R. F., & Leykin, Y. (2018). Depression increases subjective stigma of chronic pain. *Journal of Affective Disorders, 229*, 456–462. | Understand experience of depression and chronic pain and their social consequences | United States | Other | Quantitative | General |  |  |  | x |  |  |
| 58 | 44. Nguyen, A. T., Nguyen, T. H. T., Nguyen, T. T. H., Nguyen, H. T. T., Nguyen, T. X., Nguyen, T. N., ... & Vu, H. T. T. (2021). Chronic pain and associated factors related to depression among older patients in Hanoi, Vietnam. *International Journal of Environmental Research and Public Health*, *18*(17), 9192. | Investigate the frequency of chronic pain and depression among older patients and other correlated factors | Vietnam | Other | Quantitative | General |  |  |  |  | x |  |
|  | 45. Palacios-Ceña, D., Albaladejo-Vicente, R., Hernández-Barrera, V., Lima-Florencio, L., Fernández-de-Las-Peñas, C., Jimenez-Garcia, R., López-de-Andrés, A., de Miguel-Diez, J., & Perez-Farinos, N. (2021). Female Gender Is Associated with a Higher Prevalence of Chronic Neck Pain, Chronic Low Back Pain, and Migraine: Results of the Spanish National Health Survey, 2017. *Pain Medicine (Malden, Mass.), 22*(2), 382–395. | Assess the prevalence of migraine and chronic neck and low back pain, and identify socio-demographic and health-related variables associated with them | Spain | Medical/ clinical | Quantitative | Specific |  |  |  |  |  |  |
| 41 | 46. Pagán-Ortiz, M. E., & Cortés, D. E. (2021). Feasibility of an online health intervention for Latinas with chronic pain. *Rehabilitation Psychology*, *66*(1), 10. | Develop and evaluate an online health education intervention for Spanish-speaking Latina’s living with chronic pain | United States | Other | Mixed method | General | x |  |  | x | x | x |
| 30 | 47. Patel, M., Johnson, A. J., Booker, S. Q., Bartley, E. J., Palit, S., Powell-Roach, K., ... & Sibille, K. T. (2022). Applying the NIA Health Disparities Research Framework to Identify Needs and Opportunities in Chronic Musculoskeletal Pain Research. *The Journal of Pain*, 23(1), 25-44. | Evaluate what is known and needed to move research on chronic musculoskeletal pain among disproportionately affected populations forward | United States | Medical/ clinical | Literature review | General | x |  |  | x |  |  |
| 42 | 48. Peppard, C. S. W., Burkard, C. J., Georges, J., & Dye, C. J. (2022). The lived experience of military women with chronic pain: A phenomenological study. *Military Medicine*. | Examine daily life experiences among military women living with chronic pain | United States | Medical/ clinical | Qualitative | General | x |  |  | x |  | x |
| 54 | 49. Perry, M. A., Devan, H., Davies, C., Hempel, D., Ingham, T., Jones, B., ... & Hale, L. (2022). iSelf-Help: a co-designed, culturally appropriate, online pain management programme in Aotearoa. *Research Involvement and Engagement*, *8*(1), 1-15. | Develop a co-designed, culturally responsive, online group-based pain management program for people with chronic pain | New Zealand | Other | Qualitative | General | x |  |  | x | x |  |
| 70 | 50. Phifer, J., Skelton, K., Weiss, T., Schwartz, A. C., Wingo, A., Gillespie, C. F., Sands, L. A., Sayyar, S., Bradley, B., Jovanovic, T., & Ressler, K. J. (2011). Pain symptomatology and pain medication use in civilian PTSD*. PAIN, 152*(10), 2233–2240. | Assess the relationship between chronic pain and post-traumatic stress disorder among impoverished and traumatized individuals | United States | Medical/ clinical | Quantitative | General |  |  |  |  |  |  |
| 43 | 51. Poleshuck, E. L., Giles, D. E., & Tu, X. (2006). Pain and depressive symptoms among financially disadvantaged women's health patients. *Journal of Women's Health (2002), 15*(2), 182–193. | Determine the frequency of comorbid depressive symptoms and pain among gynecology outpatients and evaluate their associations with functioning and abuse experiences. | United States | Other | Quantitative | General |  |  |  | x |  | x |
|  | 52. Przekop, P., Haviland, M.G., Oda, K., & Morton, K.R. (2015). Prevalence and correlates of pain interference in older adults: Why treating the whole body and mind is necessary. *Journal of Bodywork & Movement Therapies,19*(2)*,* 217-225. | Establish a pain-related interference rate in older adults, and determine the factors associated with this interference | Canada and the United States | Other | Quantitative | General |  |  |  |  |  |  |
|  | 53. Sheth, K., Ritter, P. L., Lorig, K., Steinman, L., & FallCreek, S. (2022). Remote delivery of the chronic pain self-management program using self-directed materials and small-group telephone support: a pilot study. *Journal of Applied Gerontology*, *41*(5), 1329-1335 | Study and evaluate the reach,  effectiveness, adoption, implementation, and maintenance of a remote chronic pain program | United States | Other | Quantitative | General | x |  |  |  |  |  |
| 73 | 54. St. Marie, B. (2014). Coexisting addiction and pain in people receiving methadone for addiction. *Western Journal of Nursing Research, 36*(4), 534–551. | Examine the narratives of people with chronic pain who receive methadone treatment | United States | Medical/ clinical | Qualitative | General |  |  |  |  |  |  |
| 73 | 55. Sturycz, C. A., Hellman, N., Payne, M. F., Kuhn, B. L., Hahn, B., Lannon, E. W., Palit, S., Güereca, Y. M., Toledo, T. A., Shadlow, J. O., & Rhudy, J. L. (2019). Race/Ethnicity Does Not Moderate the Relationship Between Adverse Life Experiences and Temporal Summation of the Nociceptive Flexion Reflex and Pain: Results From the Oklahoma Study of Native American Pain Risk. *The Journal of Pain, 20*(8), 941–955 | Assess temporal summation of nociceptive flexion reflex and pain in pain-free non-Hispanic whites and Native Americans | United States | Medical/ clinical | Quantitative | General |  |  |  |  |  |  |
|  | 56. Trost, Z., Sturgeon, J., Guck, A., Ziadni, M., Nowlin, L., Goodin, B., & Scott, W. (2019). Examining Injustice Appraisals in a Racially Diverse Sample of Individuals With Chronic Low Back Pain. *The Journal of Pain, 20*(1), 83–96. | Examine associations between perceived injustice and pain, disability, and depression among people with chronic low back pain | United States | Medical/ clinical | Quantitative | Specific | x |  |  | x |  |  |
| 72 | 57. Turner, B. J., Liang, Y., Simmonds, M. J., Rodriguez, N., Bobadilla, R., & Yin, Z. (2018). Randomized Trial of Chronic Pain Self-Management Program in the Community or Clinic for Low-Income Primary Care Patients. *Journal of General Internal Medicine, 33*(5), 668–677. | Develop a chronic pain self-management program reflecting community stakeholder priorities and compare outcomes from training | United States | Medical/ clinical | Quantitative | General |  |  |  |  |  |  |
|  | 58. Turner, B. J., Rodriguez, N., Bobadilla, R., Hernandez, A. E., & Yin, Z. (2020). Chronic Pain Self-Management Program for Low-Income Patients: Themes from a Qualitative Inquiry. *Pain Medicine (Malden, Mass.), 21*(2), e1–e8. | Examine factors influencing engagement, participation, learned behaviors, and functional outcomes among socio-economically disadvantaged Hispanic patients after a chronic pain self-management program | United States | Medical/ clinical | Qualitative | General | x |  |  |  |  |  |
| 64 | 59. Velez, C. M., Nicolaidis, C., Korthuis, P. T., & Englander, H. (2017). "It's been an Experience, a Life Learning Experience": A Qualitative Study of Hospitalized Patients with Substance Use Disorders. *Journal of General Internal Medicine, 32*(3), 296–303. | Explore experiences of hospitalized adults with substance use disorder and understand factors impacting readiness for change | United States | Medical/ clinical | Qualitative | General |  |  |  | x |  |  |
| 66 | 60. Vogel, M., Choi, F., Westenberg, J. N., Cabanis, M., Nikoo, N., Nikoo, M., ... & Krausz, M. (2022). Chronic pain among individuals experiencing homelessness and its interdependence with opioid and other substance use and mental illness. *International Journal of Environmental Research and Public Health*, *19*(1), 5. | Examine the association between chronic pain and substance use among people experiencing homelessness and living with mental illness | Canada | Other | Quantitative | General |  |  |  |  |  |  |
| 60 | 61. Voon, P., Greer, A. M., Amlani, A., Newman, C., Burmeister, C., & Buxton, J. A. (2018). Pain as a risk factor for substance use: a qualitative study of people who use drugs in British Columbia, Canada. *Harm Reduction Journal*, 15(1), 35. | Explore perspectives on pain management among people who use drugs in urban and rural settings using the patient-centered care and Rhodes’ Risk Environment frameworks. | Canada | Medical/ clinical | Qualitative | General | x |  |  | x |  |  |
| 44 | 62. Walker, J. L., Harrison, T. C., & Hendrickson, S. G. (2013). Life Course Experiences, Pain and Suffering: A Case Study of an Older Mexican American Woman with Mobility Impairment. *Hispanic health care international: the official journal of the National Association of Hispanic Nurses*, 11(2), 53–61. | Understand pain and suffering, as described by an aging Mexican American woman with early onset mobility impairment, from a life course perspective | United States | Medical/ clinical | Qualitative | General | x |  |  | x | x | x |
| 31 | 63. Walker, N., Beek, K., Chen, H., Shang, J., Stevenson, S., Williams, K., ... & Cullen, P. (2022). The experiences of persistent pain among women with a history of intimate partner violence: a systematic review. *Trauma, Violence, & Abuse*, *23*(2), 490-505. | Identify studies examining experiences of  chronic pain among women who have experienced intimate partner violence. | Not applicable | Other | Systematic review | General | x |  |  | x |  | x |
| 10 | 64. Wallace, B., Varcoe, C., Holmes, C., Moosa-Mitha, M., Moor, G., Hudspith, M., & Craig, K. D. (2021). Towards health equity for people experiencing chronic pain and social marginalization. *International Journal for Equity in Health*, 20(1), 1-13. | Examine experiences of pain, stigma and discrimination in marginalized communities to recommend equity-oriented healthcare approaches to chronic pain | Canada | Other | Qualitative | General | x |  |  | x |  |  |
| 57 | 65. Walsh, K. T., Boring, B. L., Nanavaty, N., Guzman, H., & Mathur, V. A. (2022). Sociocultural context and pre-clinical pain facilitation: multiple dimensions of racialized discrimination experienced by Latinx Americans are associated with enhanced temporal summation of pain. *The Journal of Pain*, *23*(11), 1885-1893. | Focus on experiences of Latinx Americans in Texas by assessing dimensions of racialized discrimination and a laboratory marker of central pain sensitization | United States | Medical/ clinical | Quantitative | General | x |  |  | x | x |  |
| 45 | 66. Washington-Walker, J., Moore, C. L., Whittaker, T. T., & Wagner, M. L. (2017). Predictors of Medical and Vocational Rehabilitation Treatment Compliance Among African Americans with Chronic Pain Conditions: An Exploratory Study. *Journal of Applied Rehabilitation Counseling, 48*(4), 7-17. | Explore potential predictors of rehabilitation treatment compliance among African Americans receiving chronic pain management and state vocational rehabilitation agency sponsored services | United States | Medical/ clinical | Quantitative | General | x |  |  | x | x |  |
| 55 | 67. Wee, L. E., Sin, D., Cher, W. Q., Li, Z. C., Tsang, T., Shibli, S., & Koh, G. (2017). "I'm healthy, I don't have pain"- health screening participation and its association with chronic pain in a low socioeconomic status Singaporean population. *The Korean Journal of Pain, 30*(1), 34–43. | Determine the association between chronic pain and participation in health screening within a low socioeconomic-status community | Singapore | Medical/ clinical | Mixed method | General |  |  |  | x | x |  |
